# Multivalent Glycopolymer Design Unlocks Antimicrobial Activity of 2‐Deoxyglucose

**DOI:** 10.1002/anie.8647127

**Published:** 2026-04-09

**Authors:** Sungjin Jeon, Xianjin Qin, Marjon Zamani, Ariel L. Furst, Cassandra E. Callmann

**Affiliations:** ^1^ Department of Chemistry The University of Texas at Austin Austin USA; ^2^ Department of Chemical Engineering Massachusetts Institute of Technology Cambridge USA

**Keywords:** 2‐deoxyglucose, glycopolymers, multivalence, metabolic inhibition

## Abstract

With the increasing prevalence of multidrug‐resistant (MDR) pathogens, last‐line therapeutics such as cyclic peptides are insufficient, underscoring the need for new classes of antimicrobial compounds with distinct mechanisms of action. Here, we report a novel approach to glycan‐derived antimicrobials that transforms 2‐deoxyglucose (2DG), benign small molecules, into a potent antibiotic through its multivalent display as a glycopolymer. Toward this end, we synthesized a 2DG derivative amenable to ring opening metathesis polymerization (ROMP) and evaluated the impact of both spacing between 2DG and polymer backbone, as well as degree of polymerization. Shorter linkers and lower degrees of polymerization yielded the most potent antimicrobial polymer, hereafter referred to as poly2DG. Significantly, poly2DG exhibits broad‐spectrum bacterial inhibition, including against MDR methicillin‐resistant *Staphylococcus aureus* (MRSA), *Pseudomonas aeruginosa*, and *Acinetobacter baumannii*, with MIC_50_ values as low as 0.2 µg/mL. No such effect is observed for “free” 2DG nor the polymer scaffold alone, underscoring the importance of multivalent presentation for 2DG antibiotic activity. Altogether, this work shows the ability to convert an inert small molecule into an antimicrobial agent by simple polymeric scaffolding is a straightforward and effective chemical approach to develop materials that circumvent MDR.

## Graphical abstract

New approaches to combat antimicrobial‐resistant pathogens are needed. Here, scaffolding a nonmetabolizable sugar (2DG) on a polymer backbone is shown to significantly improve its antimicrobial activity.

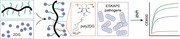

## 1 Introduction

Microbial infections are an urgent global challenge, as they often show multidrug resistance (MDR), which limits treatment options and increases the risk of mortality [1, 2, 3]. Given the rapid rise in MDR across microbial strains [4], alongside the glacial pace of antibiotic discovery, mortalities from these infections are expected to reach tens of millions in the next five years [5]. Without the immediate development of novel antimicrobials, we will return to a “pre‐antibiotic” era, in which commonplace infections are deadly [6]. Therefore, novel chemical strategies to address MDR that are not reliant on the development of novel antibiotics are urgently needed.

Among the most concerning MDR microbes are the ESKAPE pathogens: [7, 8] *Enterococcus faecium*, *Staphylococcus aureus*, *Klebsiella pneumoniae*, *Acinetobacter baumannii*, *Pseudomonas aeruginosa*, and *Enterobacter* species. These microbes are responsible for most hospital‐acquired infections [9, 10] and are often resistant to last‐line therapeutics [11], many of which are cyclic peptide macromolecules. However, peptide‐based interactions remain a small component of host‐pathogen interactions, and glycan interactions are increasingly linked to pathogenesis [12, 13] as we learn more about these biomolecules. Carbohydrate functionalized polymers, or glycopolymers, have emerged as promising alternative antimicrobial scaffolds, as they can interface with biological systems via multivalent interactions [14, 15]. Their use as antimicrobial compounds has traditionally focused on surface interactions, including preventing biofilm formation and bacterial lectin targeting [16, 17]. However, the effective dosages required for these applications remain high relative to conventional antibiotics [18, 19]. Moreover, as these materials target surface‐level interactions, they are limited in their ability to address established infections [20], which are what drive lethality.

Because glucose metabolism is essential across species, loss of efficient transport systems leaves bacteria less equipped to sustain growth, especially in biofilms. Although 2deoxyglucose, an analogue of glucose, (2DG)‐ has been extensively studied in mammalian systems, its potential as an antimicrobial has been largely overlooked. Given that glucose availability regulates virulence and biofilm formation in MDR strains including *P. aeruginosa* [21], an alternative strategy to combat MDR could be to target bacterial metabolism. 2DG, is actively transported into bacterial cells by glucose‐specific phosphotransferase systems (PTS) [22]. Once internalized, it is phosphorylated to a nonmetabolizable intermediate that depletes cellular energy reserves and stalls growth. Unlike metabolizable sugars, 2DG directly impacts cell activity by impacting this native bacterial metabolic function. Although resistance can arise through transporter loss or switching [22], these changes reduce glucose uptake efficiency and impair metabolism, particularly in nutrient‐limited environments like biofilms. Aside from isolated reports of modest inhibition in *Escherichia coli* biofilms [23], the molecule is largely inactive as a small‐molecule antibacterial agent.

Glycopolymers broadly have been shown to be antimicrobial, but these materials continue to require high dosages relative to conventional antibiotics, despite their multivalency. We hypothesized, therefore, that incorporating metabolically‐inhibitory sugars with multivalent scaffolds could support a system where the whole is greater than the sum of its parts [24]. We reasoned that targeting bacterial metabolism with glycopolymers decorated with 2DG could provide an alternative antimicrobial mechanism and introduce a new strategy to combat MDR. Herein, we report the design, synthesis, and analysis of polymers bearing 2DG as new antimicrobial materials. Through the appropriate chemical design, we show that we can convert a relatively benign small molecule into a potent antimicrobial agent through multivalent display, providing a pathway toward the development of novel antibiotic materials.

## 2 Results and Discussion

**SCHEME 1:**
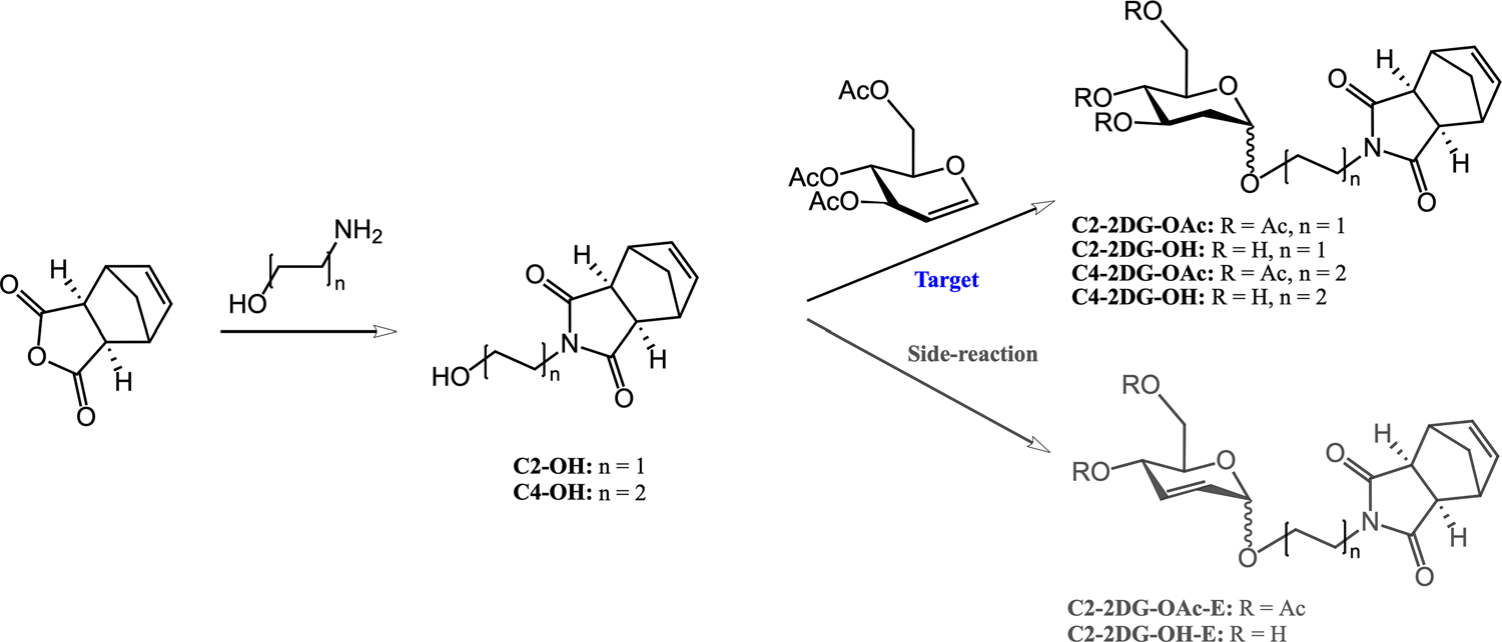
Synthetic route to the monomer series.

We designed 2DG derivatives amenable to ring opening metathesis polymerization (ROMP), with differences in the spacing between the glycan and polymerizable handle (Scheme 1), as it has been previously shown that glycan‐backbone distance can significantly influence the accessibility of the glycan and overall potency of the glycopolymer [25]. A key design criterion was to ensure that upon polymerization, these materials remain water soluble, as prior reports have indicated that polymer diffusion is significantly impeded upon gelation [26], which could further limit interactions with bacterial cells. Therefore, we chose two alkyl spacers (4‐carbon and 2‐carbon) linking the 2DG moiety to the norbornenyl handle.

To synthesize both glycomonomers, we conjugated either ethanolamine or 4‐aminobutanol to cis‐5‐Norbornene‐*exo*‐2,3‐dicarboxylic anhydride, followed by glycosylation with tri‐O‐acetylglucal. However, we observed that a Ferrier rearrangement occurred during the glycosylation reaction, resulting in a by‐product lacking the acetyl group at the C3’ position of the sugar moiety, as identified by LC‐MS analysis (Figure S1). To try to circumvent this issue, we initially attempted to use a differentially protected glucal, but saw little final product formation (Scheme S1). Therefore, we returned to our initial synthetic scheme with the tri‐O‐acetylglucal and the desired product was successfully isolated and used in subsequent reactions. We then removed the acetyl protecting groups using methoxide to afford the final glycomonomers, herein referred to as C2‐2DG‐OH and C4‐2DG‐OH.

**SCHEME 2:**
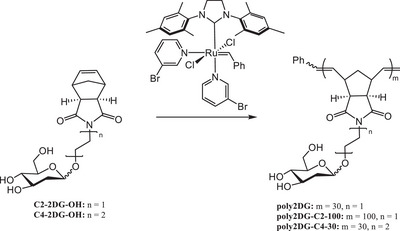
Poly2DG synthesis.

**FIGURE 1:**
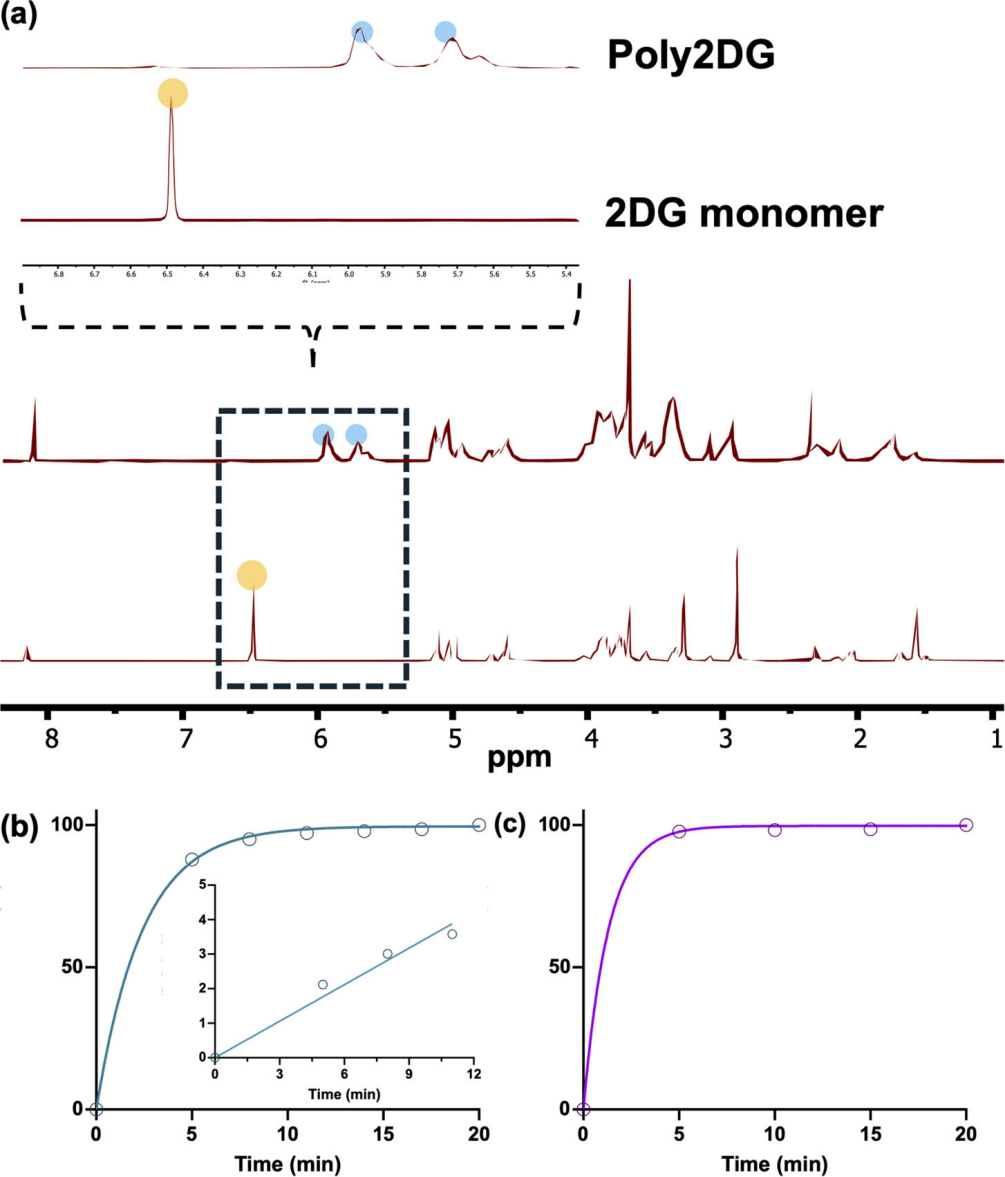
Poly2DG synthesis. (a) Representative ^1^H NMR spectra of the 2DG monomer and poly2DG, showing disappearance of olefinic protons (highlighted) upon polymerization.(b) Time‐course of C2‐2DG‐OH monomer conversion monitored by NMR, revealing rapid and complete polymerization. [Inset] First‐order kinetic plot of Poly2DG, which shows a linear relationship between ln([M]0/[M]_t_) and time and is consistent with controlled polymerization. (c) Time‐course of C4‐2DG‐OH monomer conversion monitored by NMR, revealing rapid and complete polymerization.

With both glycomonomers in hand, we next synthesized three glycopolymers that varied in either the linker length (C2 vs. C4) or degree of polymerization (30 vs. 100): poly2DG (C2‐30), poly2DG‐C2‐100, and poly2DG‐C4‐30 (Scheme 2). These variants allowed us to probe the effects of both multivalency and linker flexibility on antibiotic properties. Monomers were polymerized via ROMP using Grubbs’ third‐generation catalyst. Complete conversion of C2‐2DG‐OH was achieved within 30 min, as monitored by ^1^H‐NMR (Figure 1a) with linear kinetics (*k*_obs_ = 0.353 min^−1^, Figure 1b). Interestingly, C4‐2DG‐OH polymerized significantly faster than its shorter counterpart (Figure 1c), likely due to the increased space between the dense hydroxyl functionalities of 2DG and the ROMP handle. We characterized the resulting polymers by size exclusion chromatography coupled with multiangle light scattering (SEC‐MALS, Figures S2,S3), where we confirmed the formation of well‐defined polymers at our targeted degrees of polymerization (30 or 100, Table S1) and narrow dispersity (*Đ* < 1.20 for both polymers, see Table S1). We then confirmed that all polymers were water soluble via dynamic light scattering (DLS, Figure S4) following mild heating (37 °C). All polymers showed a hydrodynamic radius of less than 10 nm and no evidence of hydrogelation.

To both probe the ability of 2DG‐containing polymers to inhibit bacterial growth, as well as understand how linker length and degree of polymerization affect function, we next evaluated the ability of each polymer to inhibit *E. coli* growth by monitoring the change in OD600 as a function of time following polymer addition (Figure S8). Of the three polymers tested, the glycopolymer bearing a C2 spacer and DP = 30, showed the greatest suppression of bacterial growth over the course of 8 h, indicating that shorter linkers and lower degrees of polymerization enhance antimicrobial activity. Therefore, we chose this polymer to conduct further analyses, which is hereafter referred to as Poly2DG.

**FIGURE 2:**
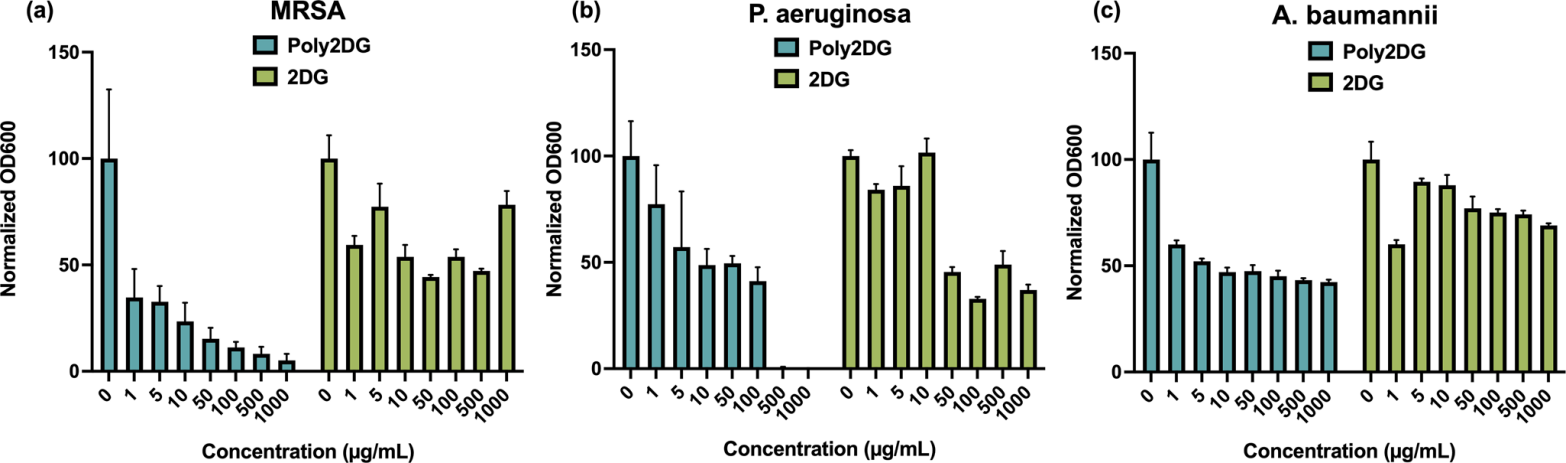
Dose‐response analysis in ESKAPE pathogens. Normalized bacterial growth (OD600) after 16 h incubation with increasing concentrations of poly2DG or free 2DG in (a) MRSA, (b) *Pseudomonas aeruginosa*, and (c) *Acinetobacter baumannii*. Poly2DG shows dose‐dependent inhibition of growth across all three clinically relevant strains, with significantly enhanced potency compared to free 2DG, which remains largely inactive even at high concentrations. These results demonstrate that multivalent presentation is essential to unlock the antibacterial potential of 2DG.

To probe the efficacy and generality of our system, we evaluated poly2DG against a broader panel of Gram‐positive and Gram‐negative bacterial species. Across all tested strains, poly2DG exhibited potent, broad‐spectrum antibacterial activity. We monitored growth inhibition over a range of poly2DG concentrations (Figure 2), which we then used to determine the MIC_50_ [20] for the polymer (Table 1). Notably, poly2DG was active against multiple pathogens, with MIC_50_ values as low as 0.2 µg/mL for MRSA and low micromolar activity observed against *P. aeruginosa* (13 ± 5 µg/mL) as well as *A. Baumannii* (17 ± 7 µg/mL). In contrast, “free” 2DG as a small molecule alone was largely inactive across all strains tested, requiring concentrations exceeding 1000 µg/mL to reach MIC_50_ in many cases.

**TABLE 1:** Antibacterial activity of poly2DG and free 2DG across a panel of Gram‐positive and Gram‐negative bacterial strains. MIC50 values represent the minimum concentration required to reduce bacterial growth by 50%, as measured by OD600. Poly2DG demonstrates potent inhibition of both Gram‐positive and Gram‐negative species, including multidrug‐resistant ESKAPE pathogens (MRSA, *P. aeruginosa*, and *A. baumannii*).

MIC50 (µg/mL)				
Bacterial Strain	Polymer	2DG	Notes	
**MRSA**	0.2 ± 0.2	74 ± 60	ESKAPE pathogen	Gram (+)
***P. aeruginosa***	13 ± 5	150 ± 50	ESKAPE pathogen	Gram (−)
***A. baumanii***	17 ± 7	>1000	ESKAPE pathogen	Gram (−)
***S. aureus***	970 ± 300	>1000	ESKAPE pathogen	Gram (+)
***B. badius***	0.6 ± 0.4	>1000	Environmental	Gram (+)
***L. fusiformis***	8.3 ± 2.8	61 ± 32	Environmental	Gram (+)
***L. innocua***	59 ± 13	330 ± 110	Listeria‐like	Gram (+)
***S. mutans***	44 ± 11	>1000	Oral pathogen	Gram (+)

Notably, we observed that the 2DG alone inhibited *P. aeruginosa* in small‐molecule form with high efficacy. This is consistent with previous studies demonstrating that the metabolic inhibition caused by this sugar leads to downstream effects beyond decreased metabolism, including inhibited biofilm formation and lower production of the phenazine pyocyanin and alginate, all of which are required for *P. aeruginosa* infection. Biofilms are required for the long‐term survival of this strain, especially in harsh or inhospitable conditions. Thus, future investigation of our multivalent scaffold will include studies of differences in the efficacy of polymeric systems in biofilm‐forming strains as compared to those that do not form biofilms, though this potential mechanistic nuance is beyond the scope of the current study.

Beyond clinically relevant strains, poly2DG also inhibited environmental and opportunistic species (Table 1, Figure S9) including *Lysinibacillus fusiformis*, *Listeria innocua*, and *Streptococcus mutans*, underscoring its broad‐spectrum potential. Interestingly, it showed reduced activity against drug‐sensitive *S. aureus* (Figure S10), suggesting that resistance‐associated metabolic states may sensitize bacteria to glycolytic interference.

To ensure that the antibiotic activity arises from 2DG and not simply a function of introducing a large macromolecule with hydrophobic domains to the bacteria [27], we synthesized a polyethylene glycol (PEG) ‐based norbornene monomer (Nor‐PEG, Scheme S4) and subsequent polymer (polyPEG, Scheme S5). As expected, polyPEG did not exhibit bacterial growth inhibition (Figure S11), indicating that the norbornene backbone introduced via ROMP does not contribute to the inhibitory effect and that bacterial inhibition is specifically attributed to the presence of densely grafted 2DG units on the polymer.

## 3 Conclusion

As hypothesized, the multivalent poly2DG demonstrated activity higher than either the backbone polymer or the 2DG alone. Though we have not yet fully elucidated the mechanism of poly2DG activity, it is clear from the broad‐spectrum microbial inhibition observed that this material serves as an important model for the continued improvement of antimicrobial glycopolymers. Specifically, by increasing the local concentration of inhibitory sugars using multivalent scaffolds, we enable their effective and efficient activity.

Collectively, these findings establish polymer‐displayed 2DG as a previously unrecognized antibacterial motif. The ability to suppress bacterial growth through metabolic interference, enabled by multivalent polymer display, offers a new direction for glycopolymer design. Our strategy expands the functional scope of sugar‐based materials from adhesion and recognition to intracellular metabolic targeting and may inform the development of polymeric agents for treating resistant infections. To our knowledge, this is the first demonstration of 2DG acting as a functional antimetabolic agent in bacteria. As such, these results establish a powerful new design strategy that integrates metabolic inhibition with materials‐based delivery, offering a new direction for glycopolymer‐based therapeutics targeting resistant and clinically relevant pathogens.

## Conflicts of Interest

The authors declare no conflicts of interest.

## Supporting information




**Supporting File**: anie71999‐sup‐0001‐SuppMat.pdf.

## Data Availability

The data that supports the findings of this study are available in the Supporting Information of this article
