# Supplementary material for: Multivalent Glycopolymer Design Unlocks Antimicrobial Activity of 2‐Deoxyglucose
Source: Angew Chem Int Ed Engl. 2026 Apr 9;65(20):e8647127. doi: 10.1002/anie.8647127 (PMC13159414; doi:10.1002/anie.8647127)
Supplement: Supplementary file 1 — Supporting File: anie71999‐sup‐0001‐SuppMat.pdf. [file ANIE-65-e8647127-s001.pdf]

Supporting Information

Multivalent Glycopolymer Design Unlocks Antimicrobial Activity of 2-Deoxyglucose

*Sungjin Jeon, Xianjin Qin, Marjon Zamani, Ariel L. Furst, and Cassandra E. Callmann**

S. Jeon, X. Qin, C. E. Callmann
Department of Chemistry, The University of Texas at Austin, Austin, TX, United States
E-mail: cassi.callmann@utexas.edu

M. Zamani, A. L. Furst
Department of Chemical Engineering, Massachusetts Institute of Technology, Cambridge, MA, United States

SUPPLEMENTAL INFORMATION
TABLE OF CONTENTS

List of Figures S3

Materials and Methods S4

Materials S4

General Methods S5

Monomer Synthesis S6

General Polymerization Protocol S13

Characterization of poly2DG by size exclusion chromatography S14

E.coli sample preparation S18

Bacterial inhibition S19

NMR spectra S21

References S37

SUPPLEMENTAL INFORMATION
LIST OF FIGURES

Figure S1. LC-MS spectrum of Ferrier rearrangement occurred product...................... S7

Figure S2. RI data of polymers. S14

Figure S3. LS data of 2DGpolymers.. S15

Figure S4. DLS analysis of polymers. S15

Figure S5. DLS characterization of poly2DG.. S16

Figure S6. DLS characterization of poly2DG-C2-100 .... S17

Figure S7. DLS characterization of poly2DG-C4-30 .... S17

Figure S8. Bacterial growth inhibition as a function of linker length and DP. S18

Figure S9. Concentration dependent on bacterial growth inhibition of bacteria S19

Figure S10. Concentration dependent on bacterial growth inhibition of *S. aureus* S20

Figure S11. Concentration dependent on bacterial growth inhibition with polyPEG S20

Materials and Methods

1. Materials

All reagents were of the highest commercial quality and used as received without further purification. Anhydrous dichloromethane (DCM) was obtained from distillation of HPLC grade dichloromethane. Cis-5-norbornene-exo-2,3-dicarboxylic anhydride was obtained from Oakwood chemical. 4-Amino-1-butanol and tri-O-acetyl-D-glucal were obtained from Ambeed. Anhydrous toluene, anhydrous N,N-dimethylformamide (DMF), ethyl vinyl ether, ethanolamine and *p*-toluenesulfonic acid monohydrate (TsOH) were obtained from sigma-aldrich. Triethylamine and sodium methoxide were obtained from fisher scientific. Silica flash column chromatography was performed using silica gel (40−63 μm), which was supplied from Sorbtech. m-PEG6-amine was obtained from broadpham. Aqueous solutions were freshly prepared with ultra-pure deionized water from a water purification system. Dialysis was performed with Snakeskin dialysis tubing, 3.5K MWCO. 16mm dry I.D. The centrifugation was performed using Sorvall X pro series centrifuge. All chemicals not mentioned were obtained from Sigma-Aldrich. Unless otherwise noted, bacteria were acquired from ATCC. The *E. coli* strain used was a gift from the Raman lab and is a derivative of K12 RL3000.

2. General Methods, Instrumentation and Measurements

Synthetic manipulations that required an inert atmosphere (where noted) were carried out under nitrogen using standard Schlenk techniques. NMR (^{1}H, ^{13}C) spectra were recorded on Bruker Prodigy 500 MHz. The ^{1}H, and ^{13}C chemical shifts were reported as δ in units of parts per million (ppm), referenced to the residual solvent. Splitting patterns are denoted as s (singlet), d (doublet), t (triplet), q (quartet), m (multiplet), and br (broad). High-resolution electrospray ionization (ESI) mass spectra were obtained at the mass spectrometry facility (the University of Texas at Austin). Size exclusion chromatography coupled with multi-angle light scattering (SEC-MALS) and size exclusion chromatography (SEC) data were measured by using TOSOH EcoSEC Elite HLC-8420GPC-Wyatt Dawn8 and and Tosoh EcoSEC HLC-8320. Bacteria were grown aerobically in incubator-shakers (New Brunswick Innova 42R). OD_{600} of cultures was monitored by UV-Visible spectroscopy using the cuvette port of a Thermo Scientific NanoDrop. High-throughput MIC_{50} assays were monitored using Agilent BioTek platereaders (Cytation and 800TS).

3. Synthesis of Compounds

3.1. Synthesis of compound C2-2DG-OH first trial

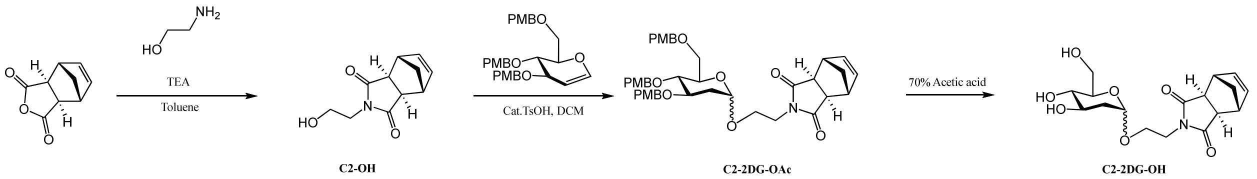

Scheme S1. Synthetic scheme for **C2-2DG-OH** first trial, reagents and conditions. (a) ethanolamine, triethylamine, toluene, reflux, 24 h, 87%. (b) tri-O-4-methoxybenzyl-D-glucal, cat. TsOH, dry DCM, 5 h. (c) 70% Acetic acid, 12 h.

3.2. Synthesis of compound C2-2DG-OH

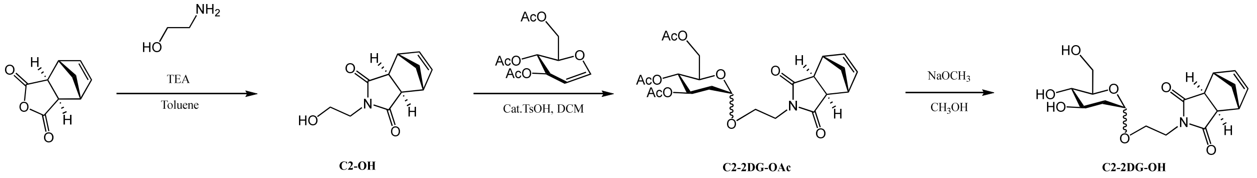

Scheme S2. Synthetic scheme for **C2-2DG-OH**, reagents and conditions. (a) ethanolamine, triethylamine, toluene, reflux, 24 h, 87%. (b) tri-O-acetyl-D-glucal, cat. TsOH, dry DCM, 5 h. (c) NaOMe, MeOH, 1 h, 37% (2 steps).

3.2.1. Compound C2-OH

5-Norbornene-2,3-dicarboxylic anhydride (5 g, 30.46 mmol) and ethanolamine (2.79 g, 45.69 mmol) were dissolved in toluene (50 mL). To the solution was added triethylamine (12.70 mL, 91.37 mmol), and then the reaction was refluxed overnight (24 h). The solution was concentrated by vacuum and the residue was purified by column chromatography on silica gel (hexane / ethyl acetate = 1:2) to afford white solid (6.6 g, 26.48 mmol, 87%). ^{1}H NMR (500 MHz, $CDCl_3$) δ 6.22 (s, 2H), 3.68 (t, J = 5.4 Hz, 2H), 3.59 (t, J = 5.3 Hz, 2H), 3.19 (s, 2H), 3.05 (s, 1H), 2.64 (s, 2H),

1.43 (d, J = 9.9 Hz, 1H), 1.28 (d, J = 9.9 Hz, 1H). ^{13}C NMR (126 MHz, $CDCl_3$) δ 178.66, 137.77, 59.68, 47.83, 45.19, 42.73, 41.13. HR-MS (ESI) Calcd for $C_{11}H_{13}NNaO_3$ $[M+Na]^+$: 230.0793, found: 230.0786.

3.2.2. Compound C2-2DG-OAc

To a stirred solution of tri-O-acetyl-D-glucal (1.36 g, 5.0 mmol) and **C2-OH** (3.11 g, 15 mmol) in dry DCM (30 mL, containing 3 Å molecular sieves) at 0 ºC cat *p*-toluenesulfonic acid (95 mg, 0.5 mmol) was added. After 7 hours the reaction at room temperature, the reaction mixture was quenched with 50 µL of TEA. The organic layer was washed three times with 50 mL brine solution, dried over Na_2SO_4, filtered, and concentrated under reduced pressure. The crude product was purified by column chromatography on silica gel with 9:1 ethyl acetate:hexanes as the mobile phase to afford **C2-2DG-OAc** as a white solid, which was used in the next step without further purification.

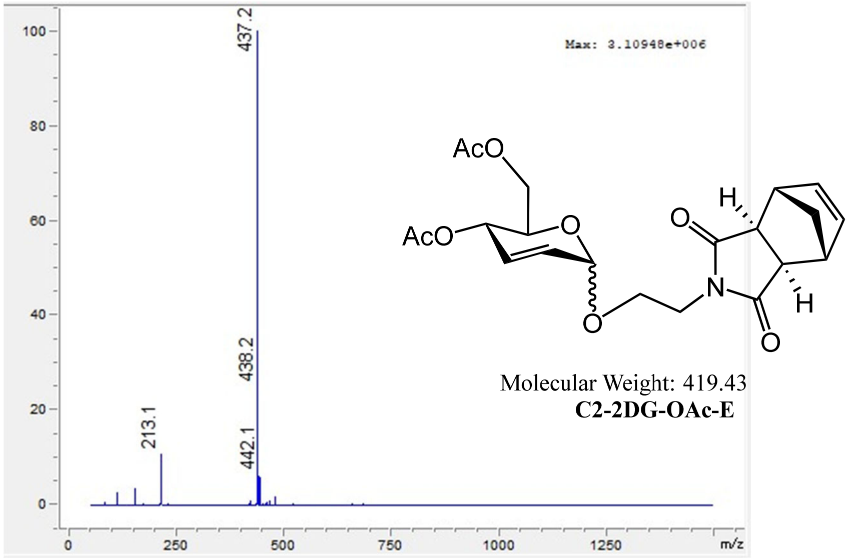

Figure S1. LC-MS spectrum of Ferrier rearrangement occurred product. Mass showed $[M+NH_4]^+$ = 437.2

3.2.3. Compound C2-2DG-OH

To a stirred solution of **C2-2DG-OAC** (1.00 g, 2.09 mmol) in MeOH (20 mL) at room temperature 0.05 eq of NaOMe (0.10 mmol) was added. After 1 hours, the reaction mixture was eluted through 2 inch silica plug using a 1:5 MeOH:DCM solution as the mobile phase. The collected solvent was concentrated under reduced vacuum then purified by column chromatography on silica gel with 1:40 to 1:20 MeOH:DCM as the mobile phase. After concentration under reduced pressure, the purified material was redissolved in acetonitrile, frozen at -80 ºC, and dried via lyophilization, yielding **C2-2DG-OH** as a white solid (0.65 g, 37%, 2 steps). ^{1}H NMR (500 MHz, CD_3OD) δ 6.33 (s, 2H), 4.90 (m, 0.67H), 4.60 – 4.55 (m, 0.38H), 3.94 (ddd, J = 12.2, 7.8, 4.8 Hz, 0.36H), 3.86 (dd, J = 11.9, 2.3 Hz, 0.36H), 3.76 (m, 3.45H), 3.65 (m, 2.76H), 3.53 (ddd, J = 12.4, 8.5, 5.0 Hz, 0.37H), 3.46 (ddd, J = 8.2, 5.5, 2.2 Hz, 0.68H), 3.19 (d, J = 7.8 Hz, 3H), 3.12 (t, J = 9.1 Hz, 0.37H), 2.73 (d, J = 5.7 Hz, 2H), 2.05 – 2.00 (m, 0.37H), 1.93 (dd, J = 13.0, 5.1 Hz, 0.68H), 1.63-1.33 (m, 3H). ^{13}C NMR (126 MHz, CD_3OD) δ 178.04, 178.00, 177.98, 177.92, 136.81, 98.29, 95.88, 75.95, 72.20, 71.18, 71.04, 70.33, 67.62, 63.22, 61.49, 60.95, 60.74, 44.37, 41.44, 41.41, 38.13, 37.02, 36.78, 36.59. HR-MS (ESI) Calc'd for $C_{17}H_{23}NNaO_7$ $[M+Na]^+$: 376.1372, found: 376.1366.

3.3. Synthesis of compound C4-2DG-OH

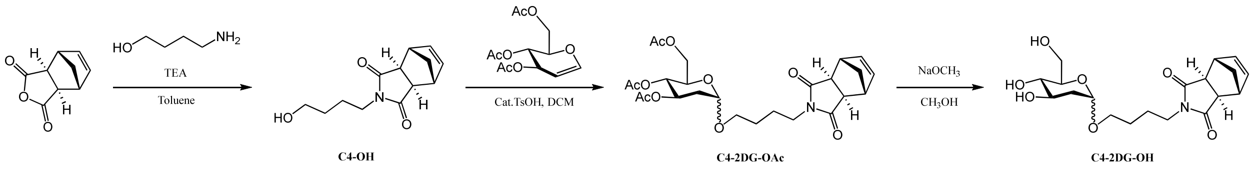

Scheme S3. Synthetic scheme for **C4-2DG-OH**, reagents and conditions. (a) 4-Amino-1-butanol, triethylamine, toluene, reflux, 24 h, 93%. (b) tri-O-acetyl-D-glucal, TsOH, dry DCM, 7 h. (c) NaOMe, MeOH, 1 h, 44% (2 steps).

3.3.1. Compound C4-OH

5-Norbornene-2,3-dicarboxylic anhydride (5 g, 30.46 mmol) and 4-aminobutan-1-ol (5.43 g, 60.92 mmol) were dissolved in toluene (50 mL). To the solution was added triethyl amine (12.70 mL, 91.37 mmol), and then the reaction was refluxed overnight. The solution was concentrated by vacuum and the residue was purified by column chromatography on silica gel (hexane / ethyl acetate = 1:2) to afford white solid (6.7 g, 28.48 mmol, 93%). ^{1}H NMR (500 MHz, $CDCl_3$) δ 6.23 (s, 2H), 3.58 (t, J = 6.3 Hz, 2H), 3.44 (t, J = 7.3 Hz, 2H), 3.20 (s, 2H), 2.62 (s, 2H), 1.58 (td, J = 8.0, 5.0 Hz, 2H), 1.53 – 1.42 (m, 3H), 1.18 – 1.14 (m, 1H). ^{13}C NMR (126 MHz, $CDCl_3$) δ 178.16, 137.77, 61.86, 47.76, 45.10, 42.68, 38.38, 29.80, 24.26. HR-MS (ESI) Calcd for $C_{13}H_{17}NNaO_3$ $[M+Na]^+$: 258.1106, found: 258.1100.

3.3.2. Compound C4-2DG-OAc

To a stirred solution of tri-O-acetyl-D-glucal (1.36 g, 5.0 mmol) and **C4-OH** (1.29 g, 5.5 mmol) in dry DCM (30 mL, containing 3 Å molecular sieves) at 0 ºC cat *p*-toluenesulfonic acid (0.5 mmol) was added. After 7 hours the reaction at room temperature, the reaction mixture was quenched with 50 µL of TEA. The organic layer was washed three times with 50 mL brine solution, dried over Na_2SO_4, filtered, and concentrated under reduced pressure. The crude product was purified by column chromatography on silica gel with 9:1 ethyl acetate:hexanes as the mobile

phase to afford **C4-2DG-OAc** as a white solid, which was used in the next step without further purification.

3.3.3. Compound C4-2DG-OH

To a stirred solution of **C4-2DG-OAC** (1.23 g, 2.43 mmol) in MeOH (20 mL) at room temperature 0.05 eq of NaOMe (0.12 mmol) was added. After 1 hours, the reaction mixture was eluted through a 2 inch silica plug using a 1:5 MeOH:DCM solution as the mobile phase. The collected solvent was concentrated under reduced vacuum then purified by column chromatography on silica gel with 1:40 to 1:20 MeOH:DCM as the mobile phase. After concentration under reduced pressure, the purified material was redissolved in acetonitrile, frozen at -80 ºC, and dried via lyophilization, yielding **C4-2DG-OH** as a white solid (0.84 g, 44%, 2 steps). ^{1}H NMR (500 MHz, CD_3OD) δ 6.37-6.32 (s, 2H), 4.89 (m, 0.56H), 4.55 (dd, J = 9.6, 1.9 Hz, 0.44H), 3.96 – 3.78 (m, 2H), 3.71 (h, J = 6.0 Hz, 1.5H), 3.45 –3.60 (m, 3.5H), 3.43 – 3.35 (m, 0.5H), 3.28 – 3.15 (m, 3.5H), 2.70-2.79 (s, 2H), 2.15 – 23.03 (m, 1H), 1.69 – 1.44 (m, 6H), 1.25 (d, J = 9.7 Hz, 1H). ^{13}C NMR (126 MHz, CD_3OD) δ 178.04, 136.80, 99.05, 96.56, 75.91, 71.93, 71.27, 71.06, 70.44, 67.85, 67.32, 65.44, 60.91, 60.79, 44.21, 41.40, 38.29, 37.25, 37.19, 36.82, 26.07, 25.96, 23.70, 23.47. HR-MS (ESI): calcd. for $C_{19}H_{27}NNaO_7$ $[M+Na]^+$ = 404.1680, found 404.1674.

3.4. Synthesis of compound Nor-PEG

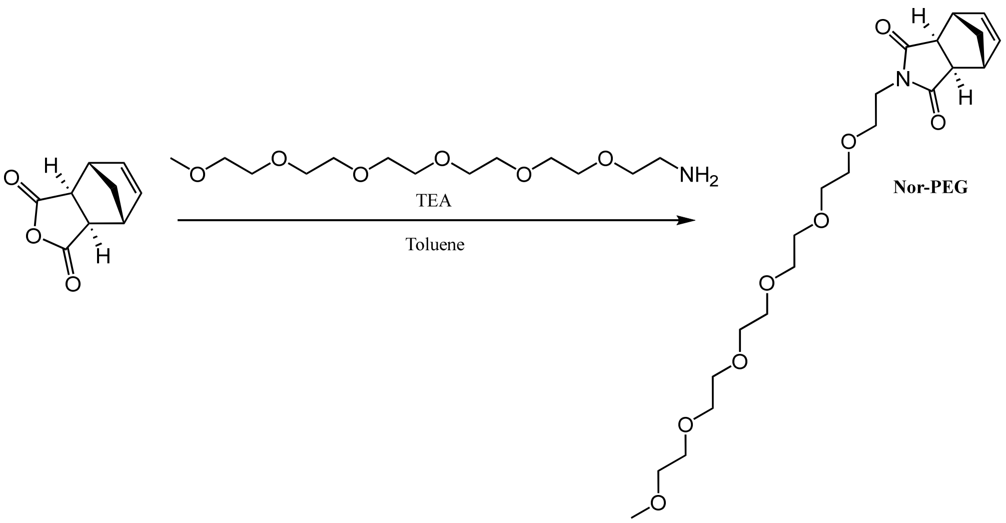

Scheme S4. Synthetic scheme for **Nor-PEG**, reagents and conditions. (a) m-PEG6-amine, triethylamine, toluene, reflux, 24 h, 87%.

3.4.1. Compound nor-PEG

5-Norbornene-2,3-dicarboxylic anhydride (1.3 g, 7.92 mmol) and m-PEG6-amine (2.0 g, 6.78 mmol) were dissolved in toluene (50 mL). To the solution was added triethyl amine (0.09 mL, 0.68 mmol), and then the reaction was refluxed overnight. The solution was concentrated by vacuum and the residue was purified by column chromatography on silica gel (hexane / ethyl acetate = 3:1) to afford white solid (2.6 g, 5.90 mmol, 87%). ^{1}H NMR (400 MHz, $CDCl_3$) δ 6.21 (t, J = 1.8 Hz, 2H), 3.66 – 3.45 (m, 24H), 3.31 (s, 3H), 3.20 (p, J = 1.7 Hz, 2H), 2.61 (d, J = 1.4 Hz, 2H), 1.42 (dt, J = 9.9, 1.7 Hz, 1H), 1.33 – 1.26 (m, 1H). ^{13}C NMR (101 MHz, DMSO-*d6*) δ 178.02, 138.12,

71.75, 70.27, 70.25, 70.13, 70.05, 69.74, 66.48, 58.52, 47.68, 45.07, 42.71, 37.88. HR-MS (ESI) Calcd for $C_{22}H_{35}NO_8$ $[M+H]^+$: 442.2435, found: 442.2442.

4. General Polymerization Procedure

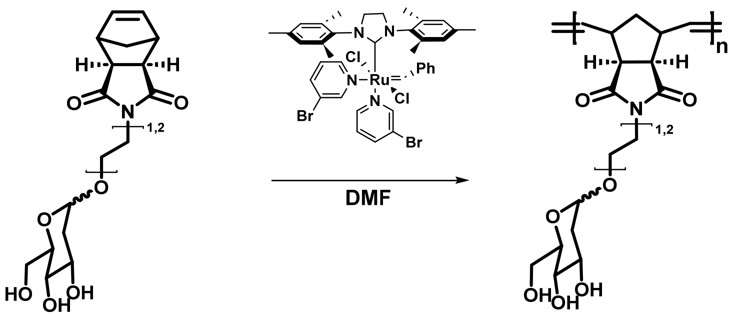

Scheme S5. General graft-through polymerization procedure.

To a 8.0 mL vial with a solution of **C2-2DG-OH**, **C4-2DG-OH** and **Nor-PEG** (50 mg, **poly2DG**: 142 µmol, 30 eq, **poly2DG-C2-100**: 142 µmol, 100 eq, **poly2DG-C4-30**: 131 µmol, 30 eq and **polyPEG**: 113 µmol, 30 eq) in dry DMF (800 µL) was added a solution of Grubbs M300® catalyst (**poly2DG**: 4.73 µmol, 1 eq, **poly2DG-C2-100**: 1.42 µmol, 1 eq, **poly2DG-C4-30**: 4.37 µmol, 1 eq and **polyPEG**: 3.78 µmol, 1 eq) in dry DMF (200 µL). The vial was quickly stirred and incubated in the glove box. This reaction was allowed to stir under N_2 for 2 hours at room temperature. The polymerization was quenched with 50 µL of ethyl vinyl ether for 10 minutes. Each polymer was analyzed using SEC-MALS. The polymers in DMF were then slowly dripped into a conical tube with 40 mL of chilled (-80 °C) diethyl ether and then centrifuged at 8,000-10,000 xg for 5 minutes. The supernatant was decanted, and the polymer pellet dried under vacuum and purified with 3K snakeskin dialysis tubing using H_2O for 2 days.

5. Characterization of poly2DG by size exclusion chromatography

Each polymer was dissolved in DMF (THF for polyPEG) at a concentration of 0.4-0.5 mg/mL, filtered through a 0.45 µm syringe filter, and injected in a volume of 50 µL. Polymerization of each sample was confirmed by ^{1}H-NMR, which showed the disappearance of the monomer double bond (~6.2 ppm) and the formation of trans/cis double bonds (5.0~5.5 ppm) characteristic of the polymer. [1, 2] Dynamic light scattering (DLS) samples were prepared by dissolving the polymers in 18 MΩ water at a concentration of 0.5 mg/mL, followed by incubation at 37 °C for 1 hour. Measurements were performed using disposable cuvettes, with three sets of 10 scans each. The results are reported as the mean ± standard deviation.

Eluent conditions:

2DG polymers (SEC-MALS): TSKgel α-M (0018344) column, 0.4 ml/min, 60 ºC, 10 mM LiBr in DMF, Wyatt DAWN 8 (WD3−03) light scattering detector.

PolyPEG (SEC): TSKgel SuperHM-H column, 0.5 ml/min, 40 ºC, HPLC grade THF, Polystyrene standard.

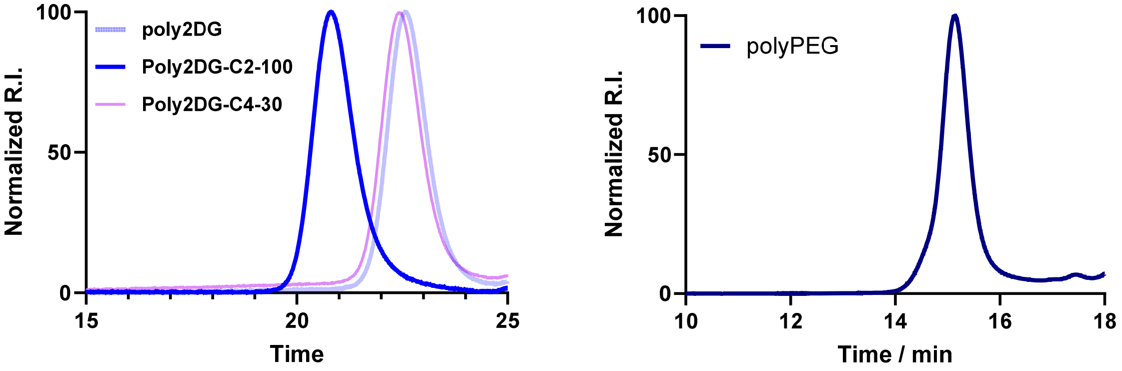

Figure S2. Refractive index (RI) data of poly2DG (light blue, left), Poly2DG-C2-100 (blue, left), Poly2DG-C4-30 (light purple, left) and SEC of polyPEG (dark blue, right).

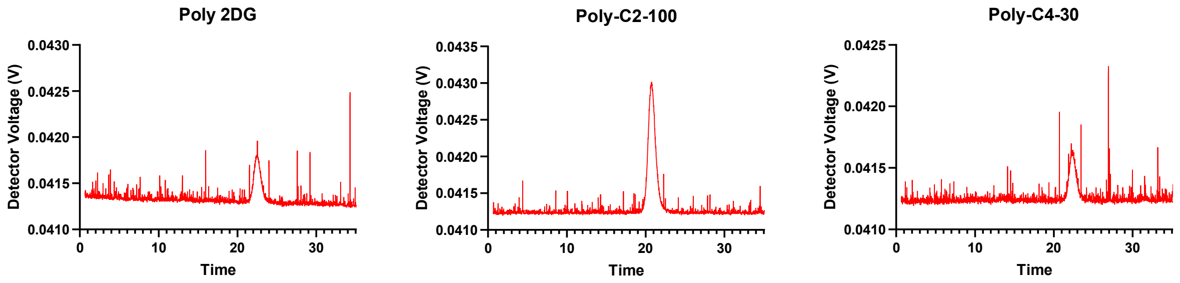

Figure S3. Light scattering (LS) data of poly2DG (left), Poly2DG-C2-100 (middle), Poly2DG-C4-30 (right).

Table S1. Polymer characterization by SEC-MALS.

Polymer	Target DP	Theo. M_n (kDa)	M_n (kDa)	M_w (kDa)	DP (M_n)	DP (NMR)	Đ
C2-30	30	10.59	9.46	9.81	28	31	1.037
C2-100	100	35.37	37.12	37.96	105	92	1.023
C4-30	30	11.43	9.93	10.27	26	30	1.034
polyPEG	30	13.23	15.07	16.87	34	31	1.12

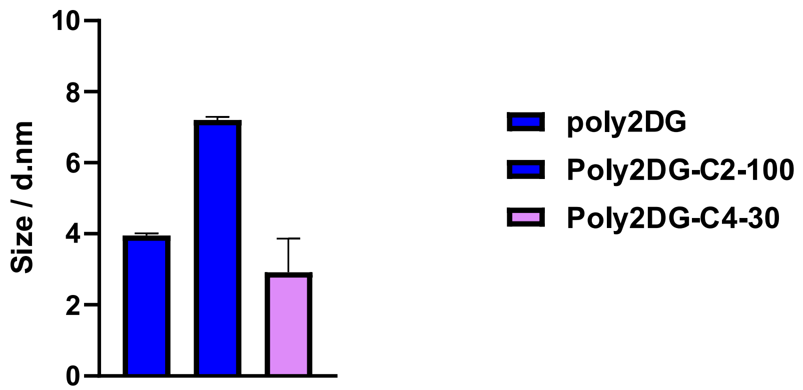

Figure S4. DLS analysis of poly2DG (light blue), Poly2DG-C2-100 (blue) and Poly2DG-C4-30 (light purple). [polymers] = 0.5 mg/mL. Particle sizes were calculated based on the Number distribution.

Table S2. Polymer size characterization by DLS.

Polymer	Size (nm)
Poly2DG	3.957 ± 0.567
Poly2DG-C2-100	7.200 ± 0.095
Poly2DG-C4-30	2.923 ± 0.937

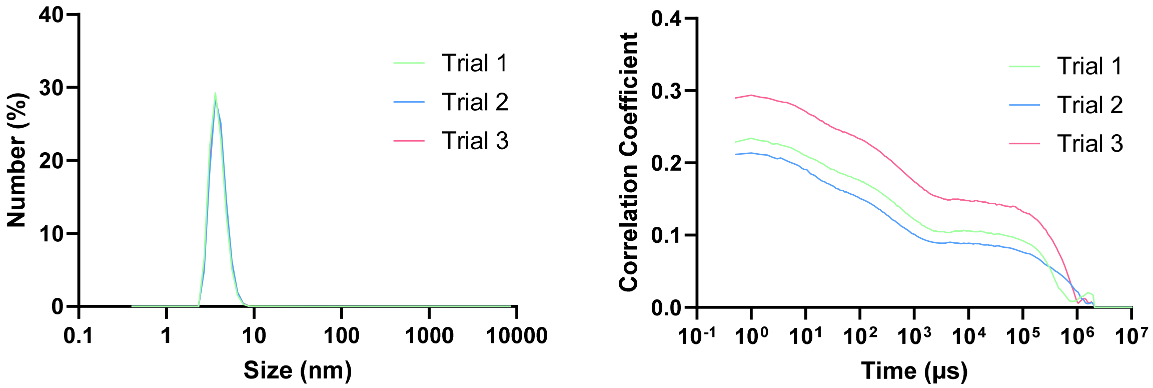

Figure S5. DLS characterization of poly2DG. Number size distribution (left) and raw correlation data (correlogram) of poly2DG (right). Each measurement was performed with 10 scans per run.

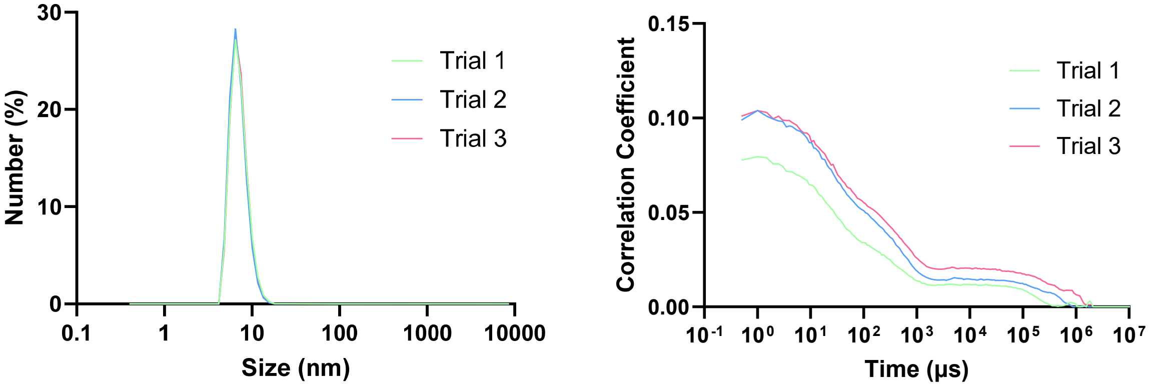

Figure S6. DLS characterization of poly2DG-C2-100. Number size distribution (left) and raw correlation data (correlogram) of poly2DG (right). Each measurement was performed with 10 scans per run.

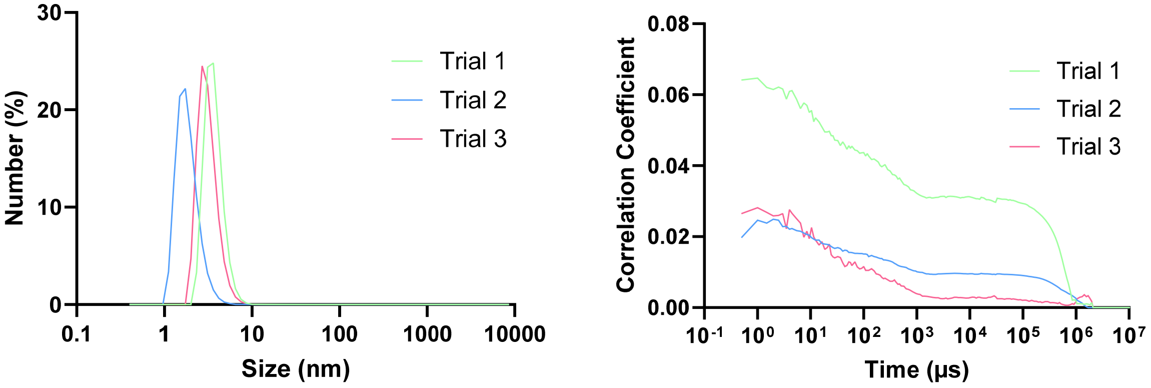

Figure S7. DLS characterization of poly2DG-C4-30. Number-weighted size distribution (left) and raw correlation data (correlogram) of poly2DG (right). Each measurement was performed with 10 scans per run.

6. *E. coli* sample preparation

E. coli was grown from individual colonies in LB at 37 ºC with shaking at 250 RPM for 12-18 h. Cells were then pelleted by centrifugation for 2 min at 10,000 RPM then resuspended in either LB rich media or M9 minimal media to a final OD_{600} of 0.1. Subsequent growth inhibition assays were performed by addition of the polymer in 1 μL DMSO to 99 μL cells in a 384-well plate. Control conditions included the addition of 1 μL DMSO. The plate was analyzed for over 10 hours with continuous incubation at 37 ºC with shaking at 250 RPM and an absorbance reading every 10 min.

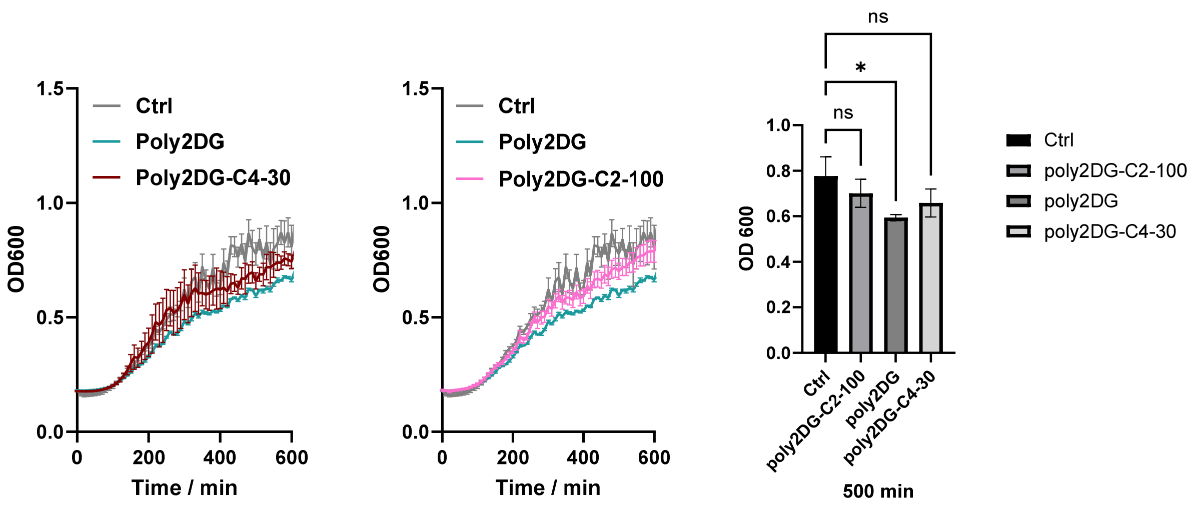

Figure S8. Bacterial growth inhibition as a function of degree of polymerization and linker length. Growth inhibition was evaluated by OD_{600} for polymers with DP 30 (left), by OD_{600} for polymers with DP 100 (middle) and growth inhibition at 500 min (right). The degree of inhibition is lower than in Section 7 (below), as these were evaluated in rich media. Each time interval = 10 min.

7. Bacterial growth inhibition

All bacteria were grown from individual colonies in their preferred rich media at either 30 ºC or 37 ºC with shaking at 250 RPM for 12-18 h. Cells were then pelleted by centrifugation for 2 min at 10,000 RPM then resuspended in either their preferred rich media or M9 minimal media to a final OD_{600} of 0.1. Subsequent growth inhibition assays were performed by addition of the polymer or monomer in 1 μL DMSO to 99 μL cells in a 384-well plate. Control conditions included the addition of 1 μL DMSO only to cells. The plates were monitored for at least 24 hours with continuous incubation at the preferred temperature of the strain with shaking at 250 RPM and an absorbance reading every 10 min.

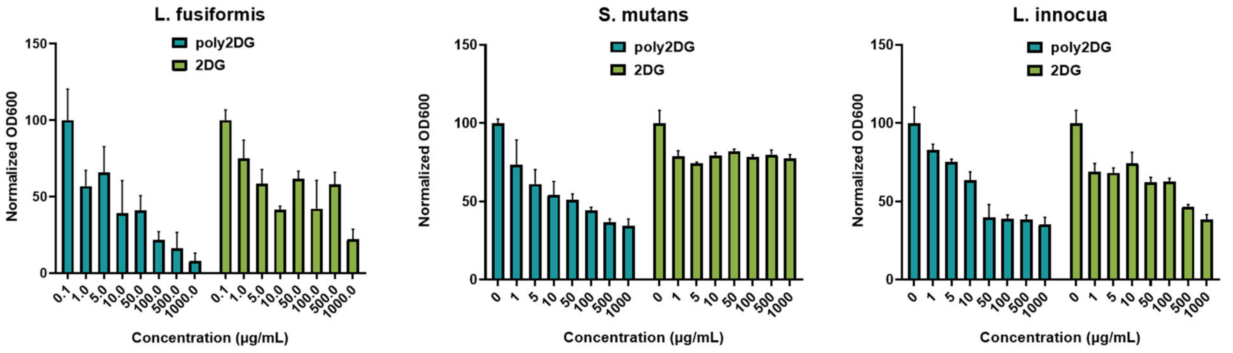

Figure S9. Concentration dependent on the bacterial growth inhibition of *L. fusiformis, L. innocua*, and *S. mutans*. *L. fusiformis* (left), *S. mutans* (middle), and *L. innocua* (right). Poly2DG (blue) and 2-deoxyglucose (green).

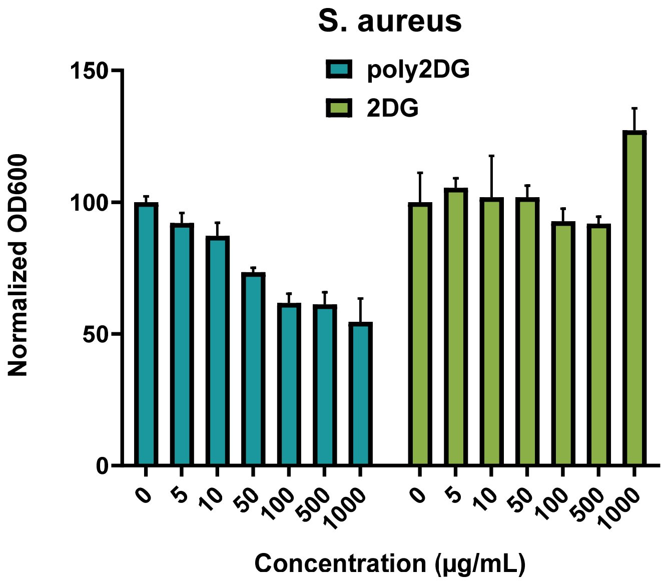

Figure S10. Concentration dependent on the bacterial growth inhibition of *S. aureus*. Poly2DG (blue) and 2-deoxyglucose (green).

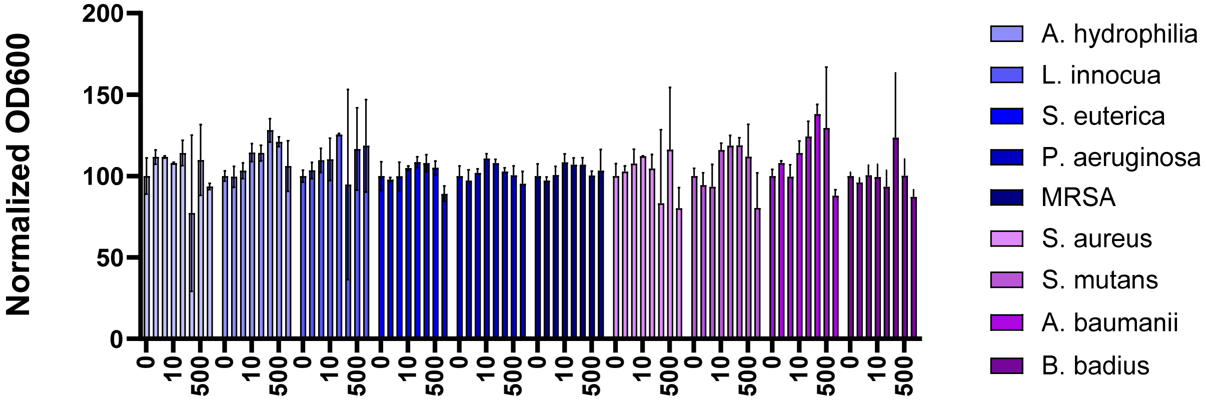

Figure S11. Concentration dependent on the bacterial growth inhibition with polyPEG.

8. ^{1}H-NMR and ^{13}C-NMR Spectra

^{1}H-NMR Spectrum of **C2-OH** in $CDCl_3$ (500 MHz):

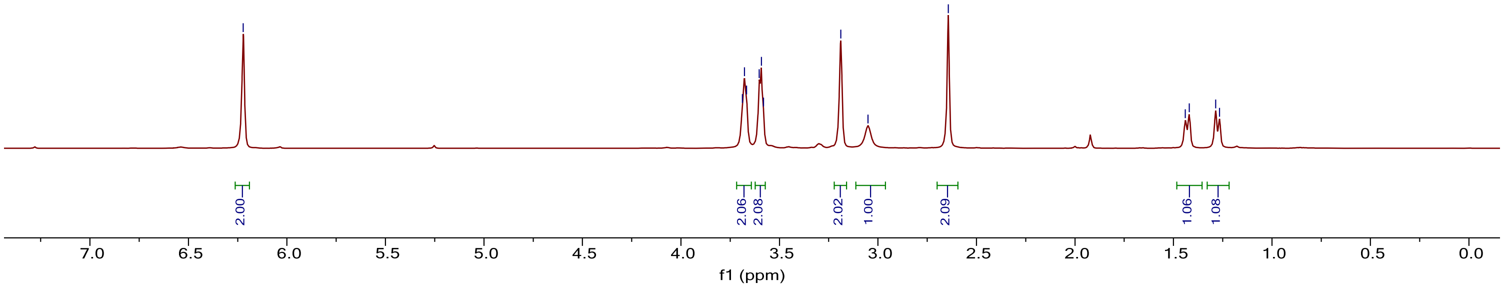

^{13}C-NMR Spectrum of **C2-OH** in $CDCl_3$ (126 MHz):

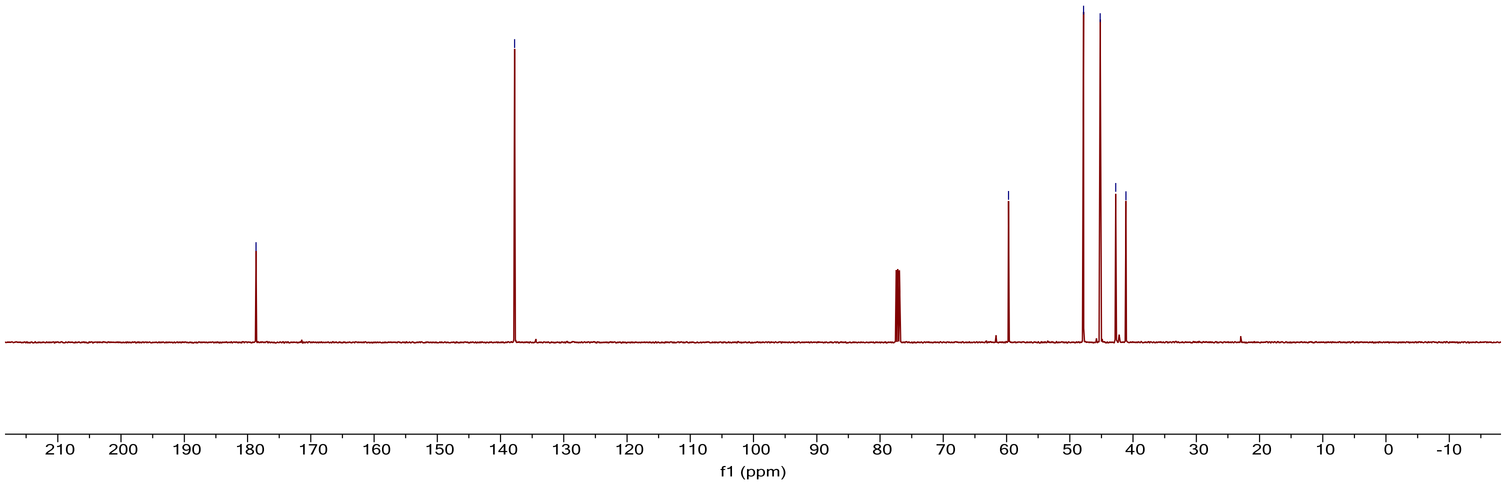

^{1}H-NMR Spectrum of **C2-2DG-OH** in CD_3OD (500 MHz):

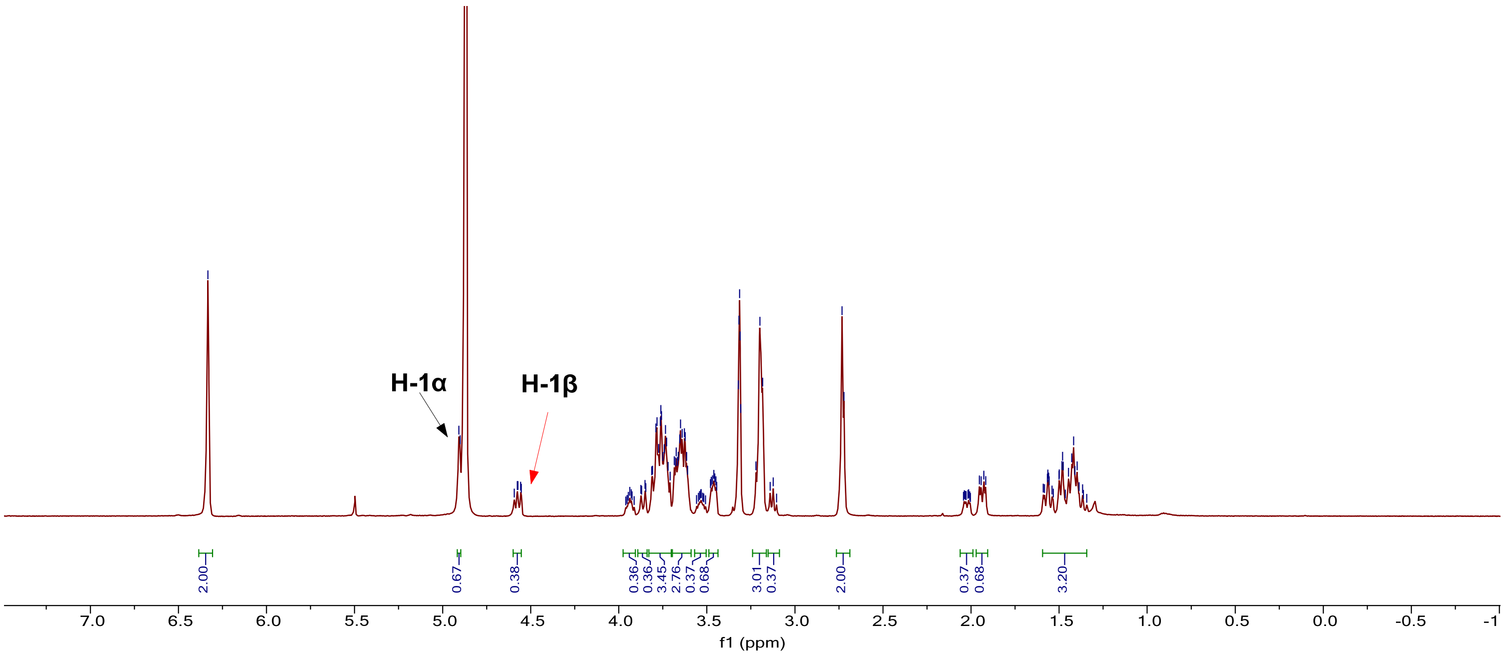

^{13}C-NMR Spectrum of **C2-2DG-OH** in CD_3OD (126 MHz):

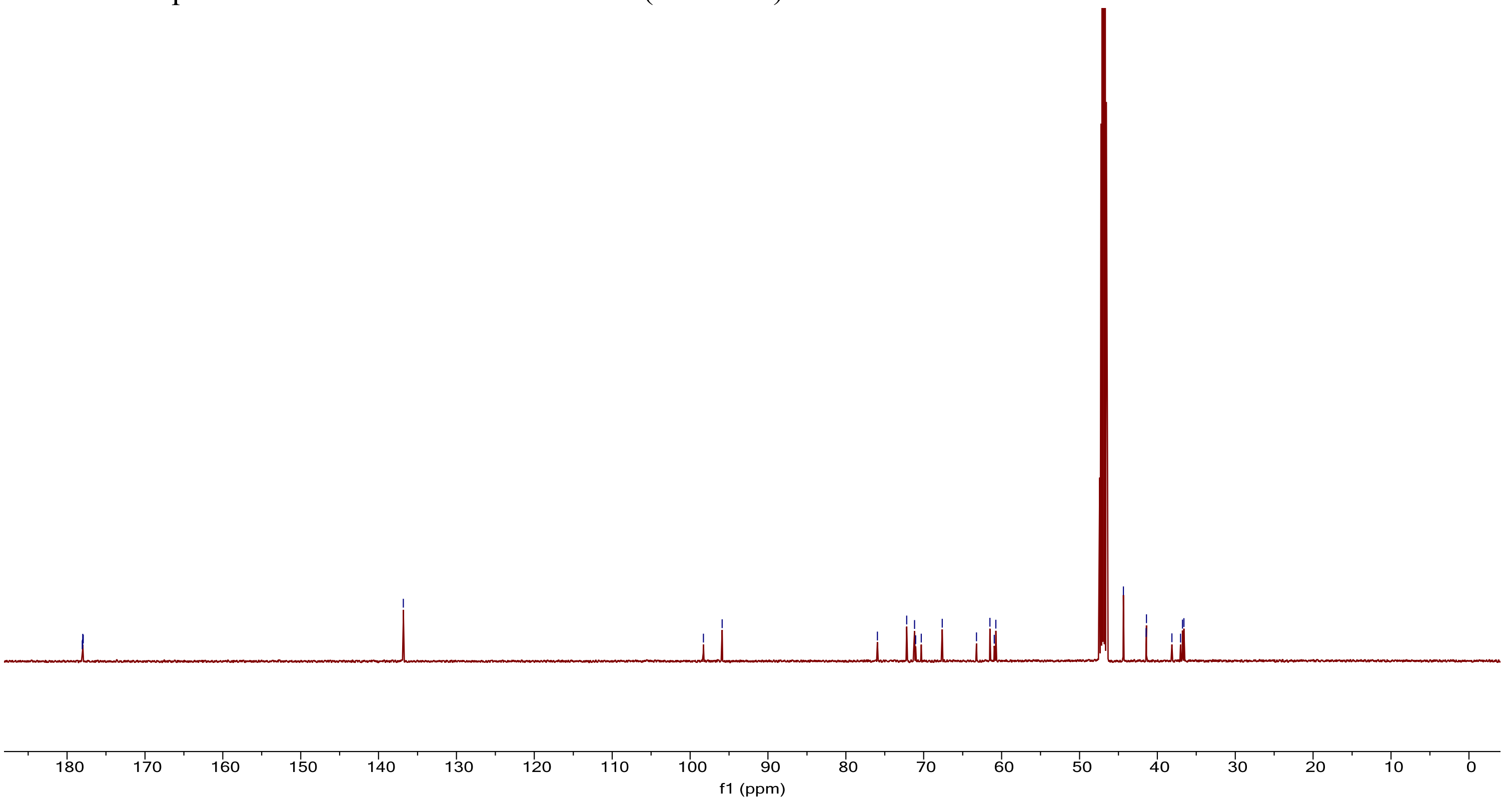

2D-HSQC Spectrum of C**2-2DG-OH** in CD_3OD:

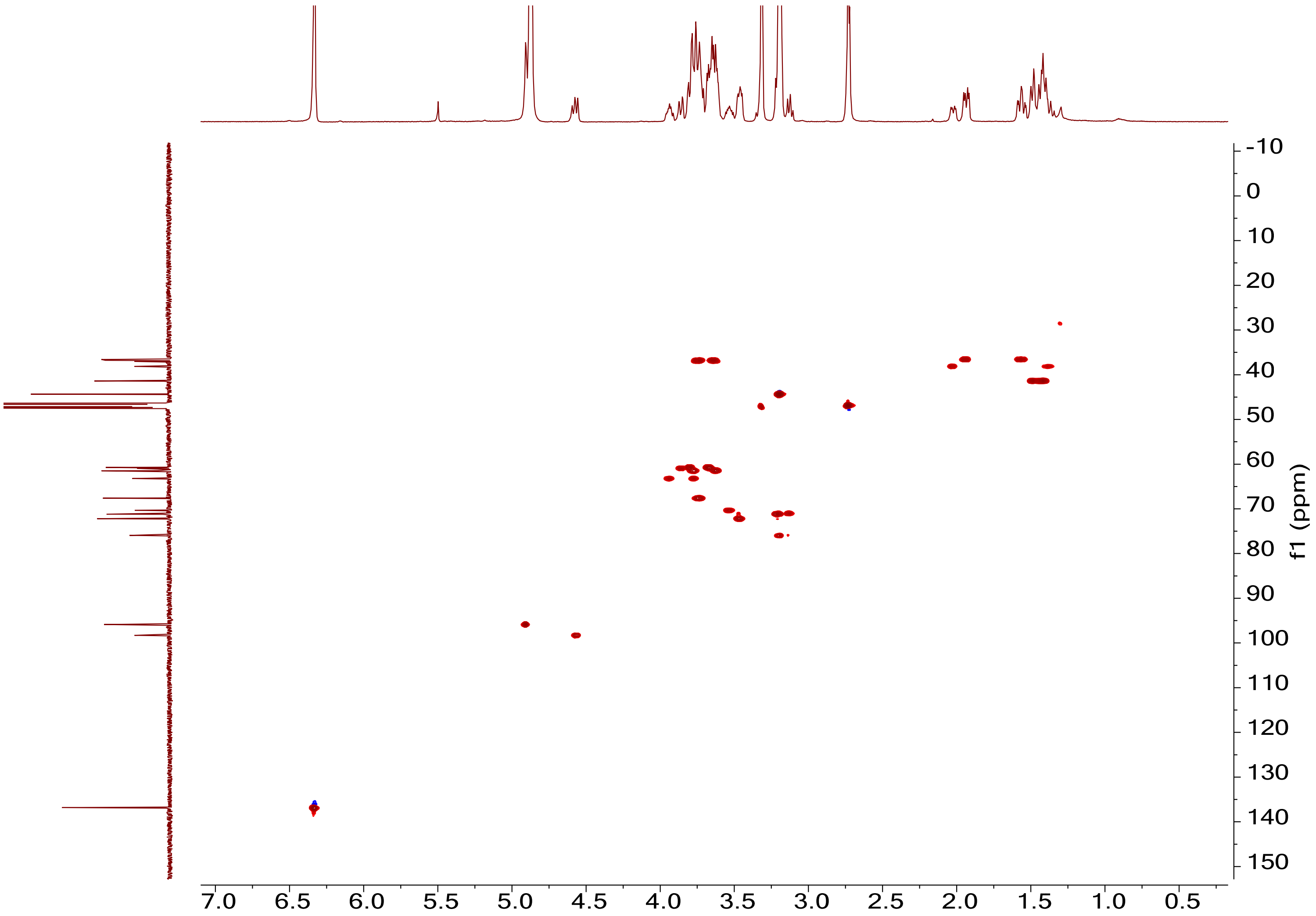

^{1}H-NMR Spectrum of **C4-OH** in $CDCl_3$ (500 MHz):

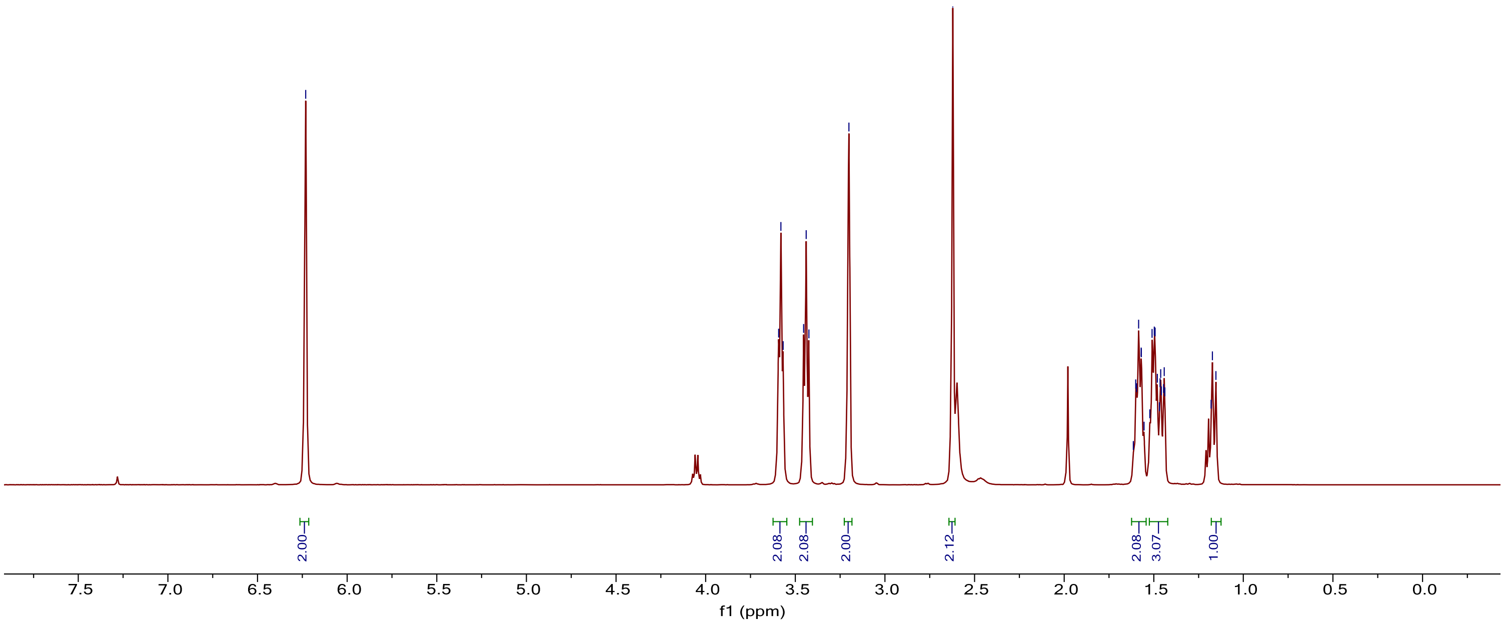

^{13}C-NMR Spectrum of **C4-OH** in $CDCl_3$ (126 MHz):

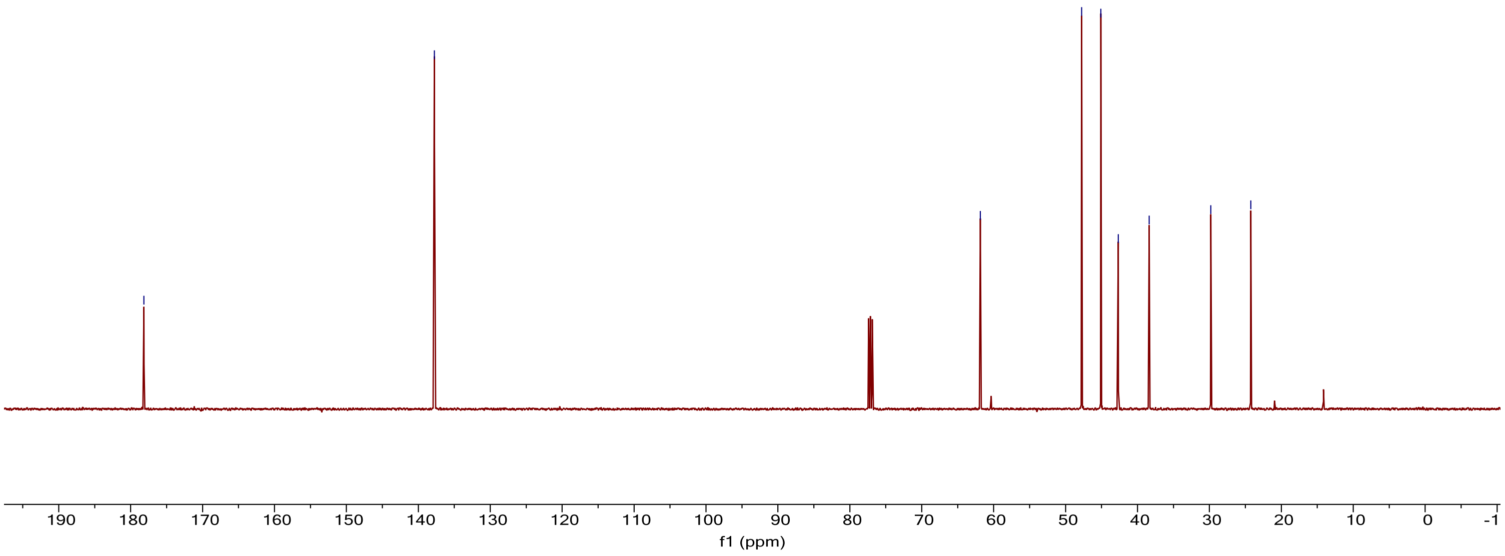

^{1}H-NMR Spectrum of **C4-2DG-OH** in CD_3OD (500 MHz):

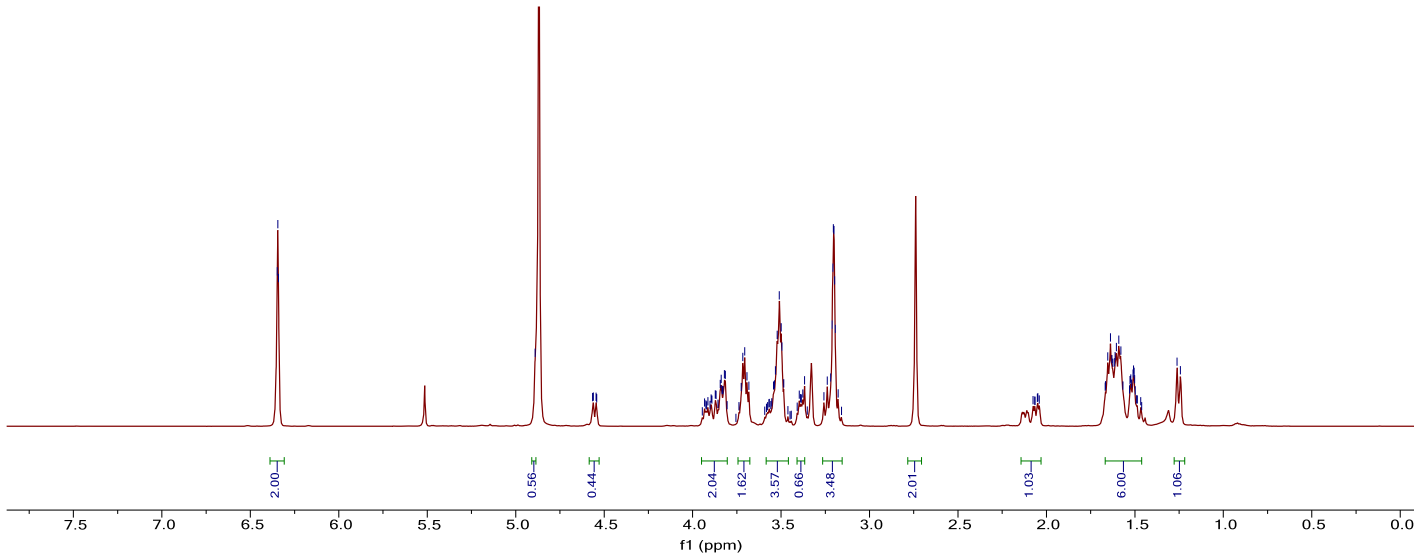

^{13}C-NMR Spectrum of **C4-2DG-OH** in CD_3OD (126 MHz):

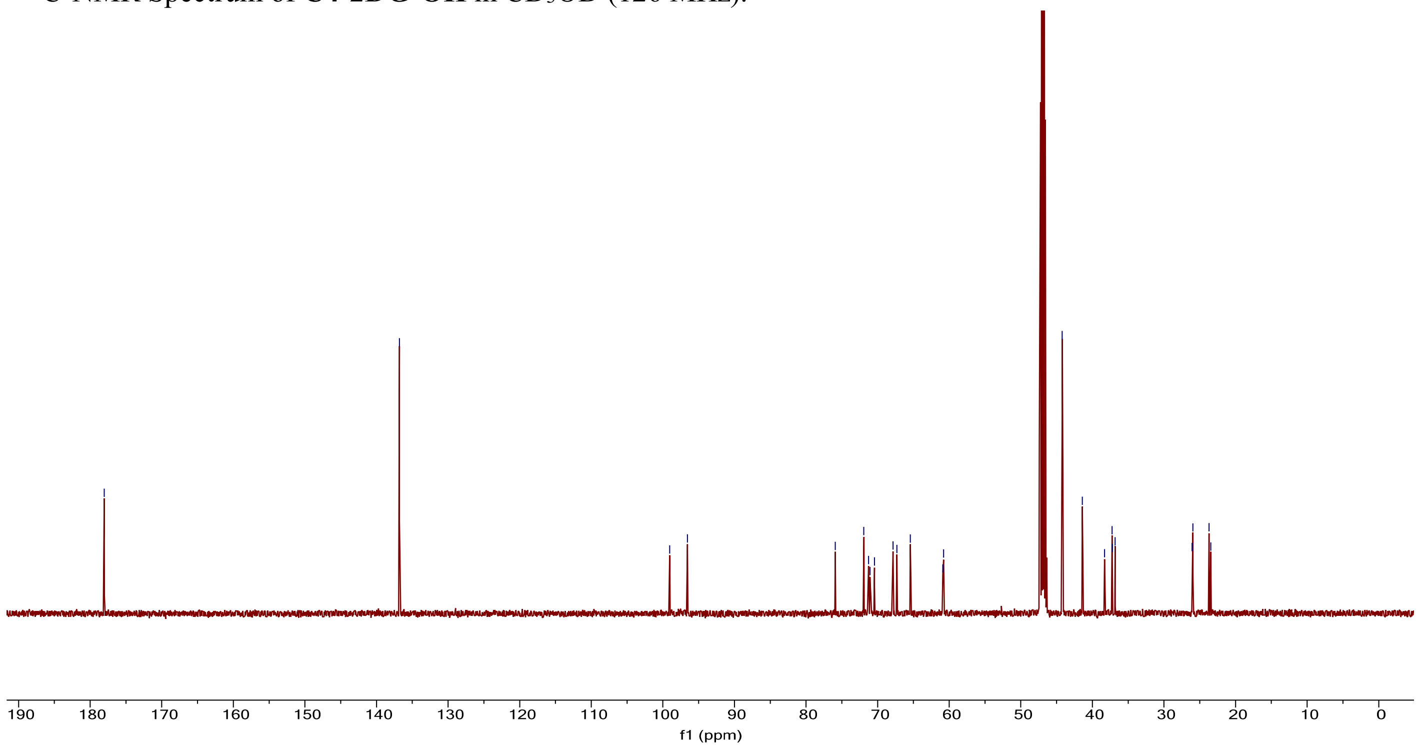

2D-HSQC Spectrum of **C4-2DG-OH** in CD_3OD:

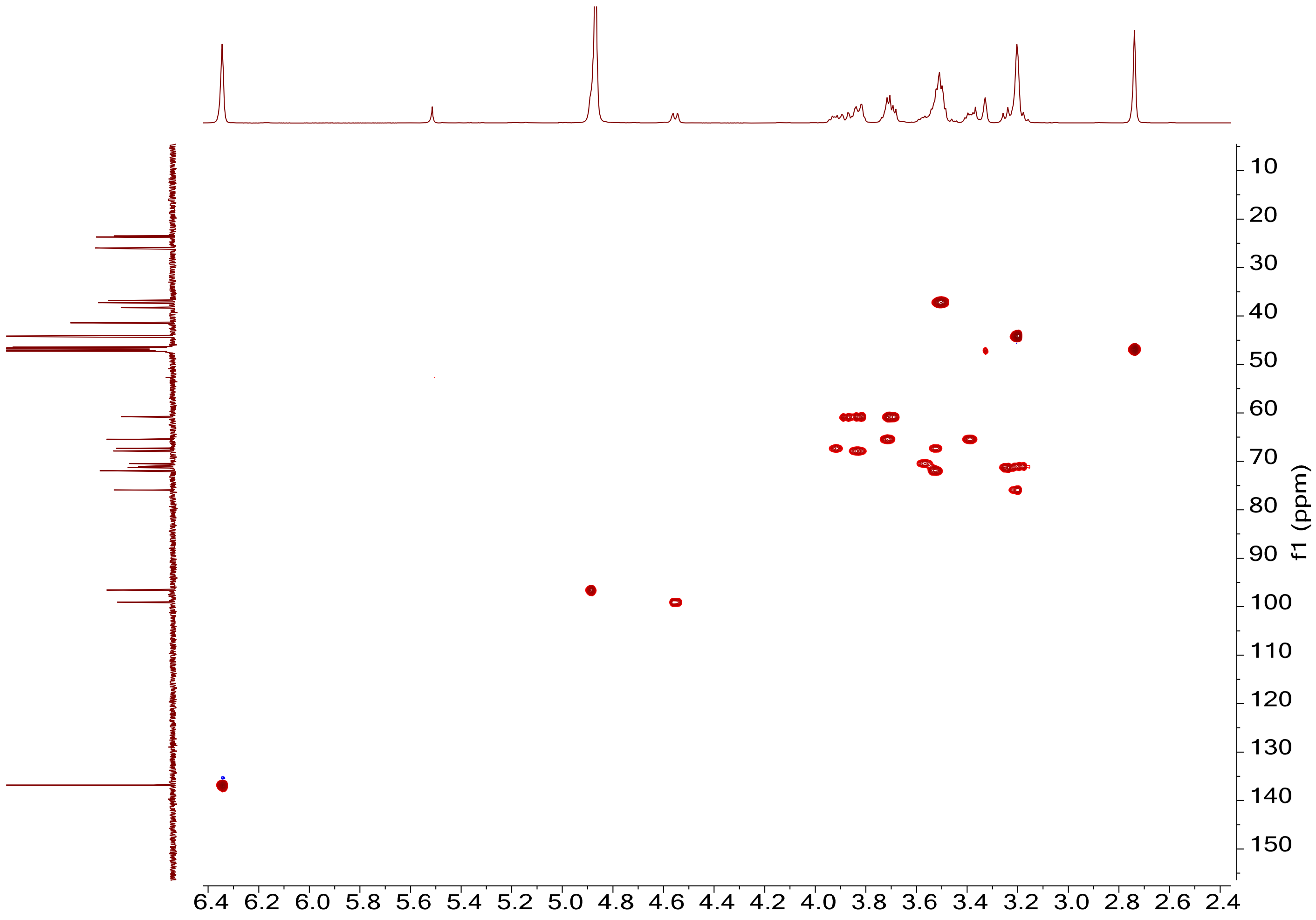

^{1}H-NMR Spectrum of **Nor-PEG** in $CDCl_3$ (400 MHz):

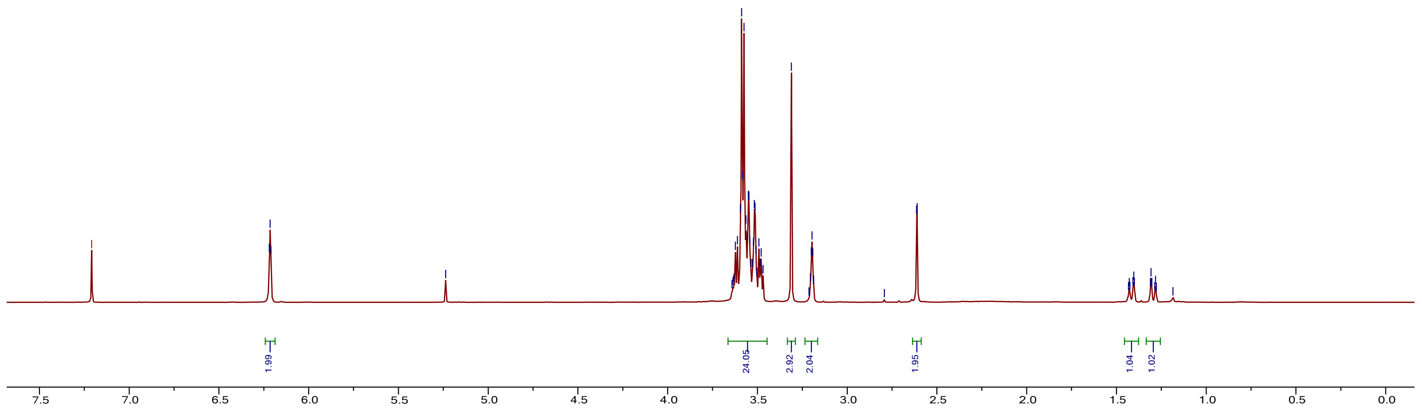

^{13}C-NMR Spectrum of **Nor-PEG** in DMSO-*d6* (101 MHz):

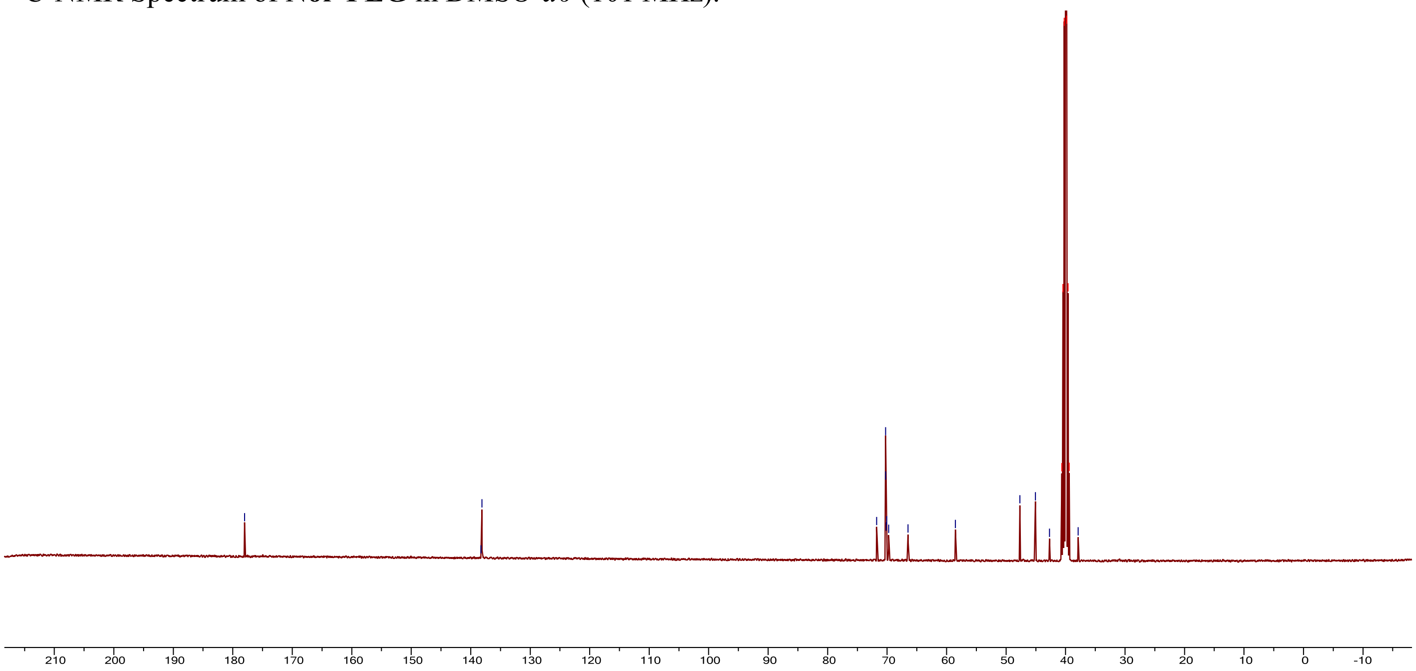

^{1}H-NMR spectra of **poly2DG** in DMSO-*d6*:

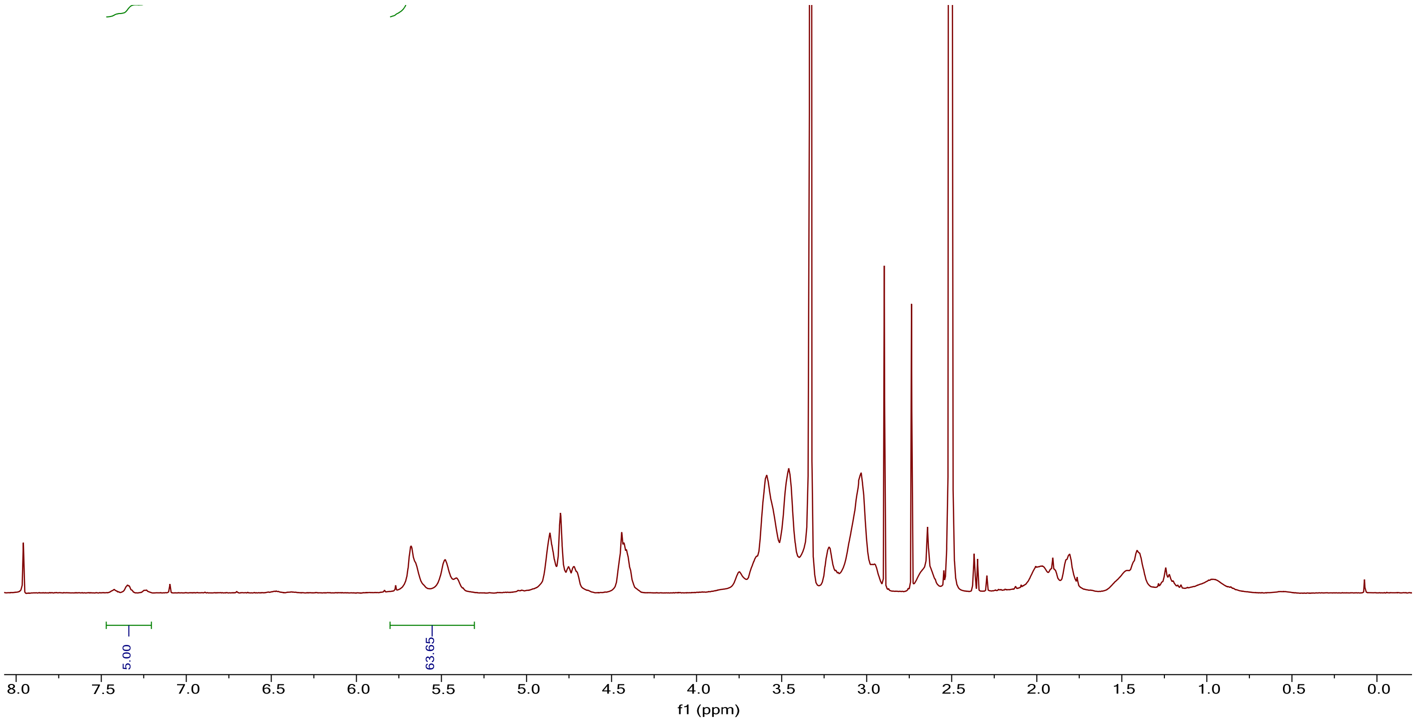

^{1}H-NMR spectra of **poly2DG-C2-100** in DMSO-*d6*:

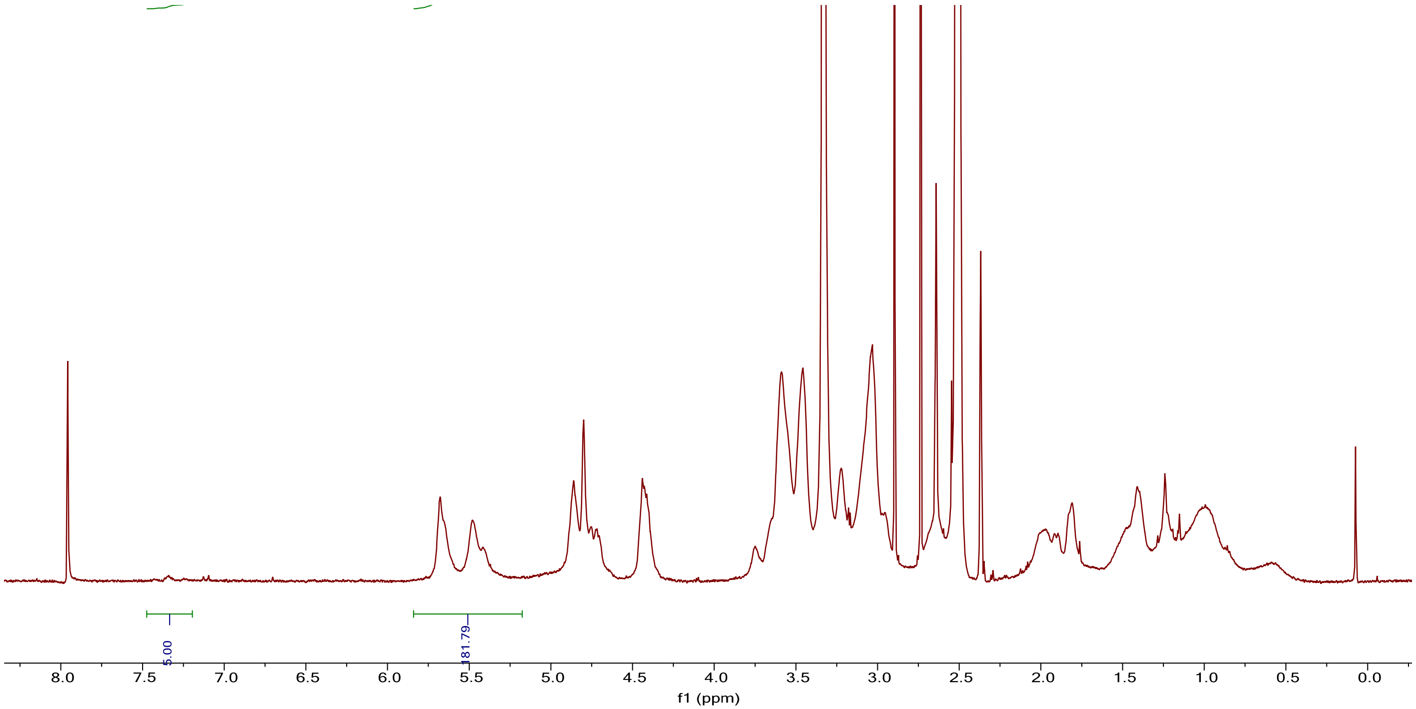

^{1}H-NMR spectra of **poly2DG-C4-30** in DMSO-*d6*:

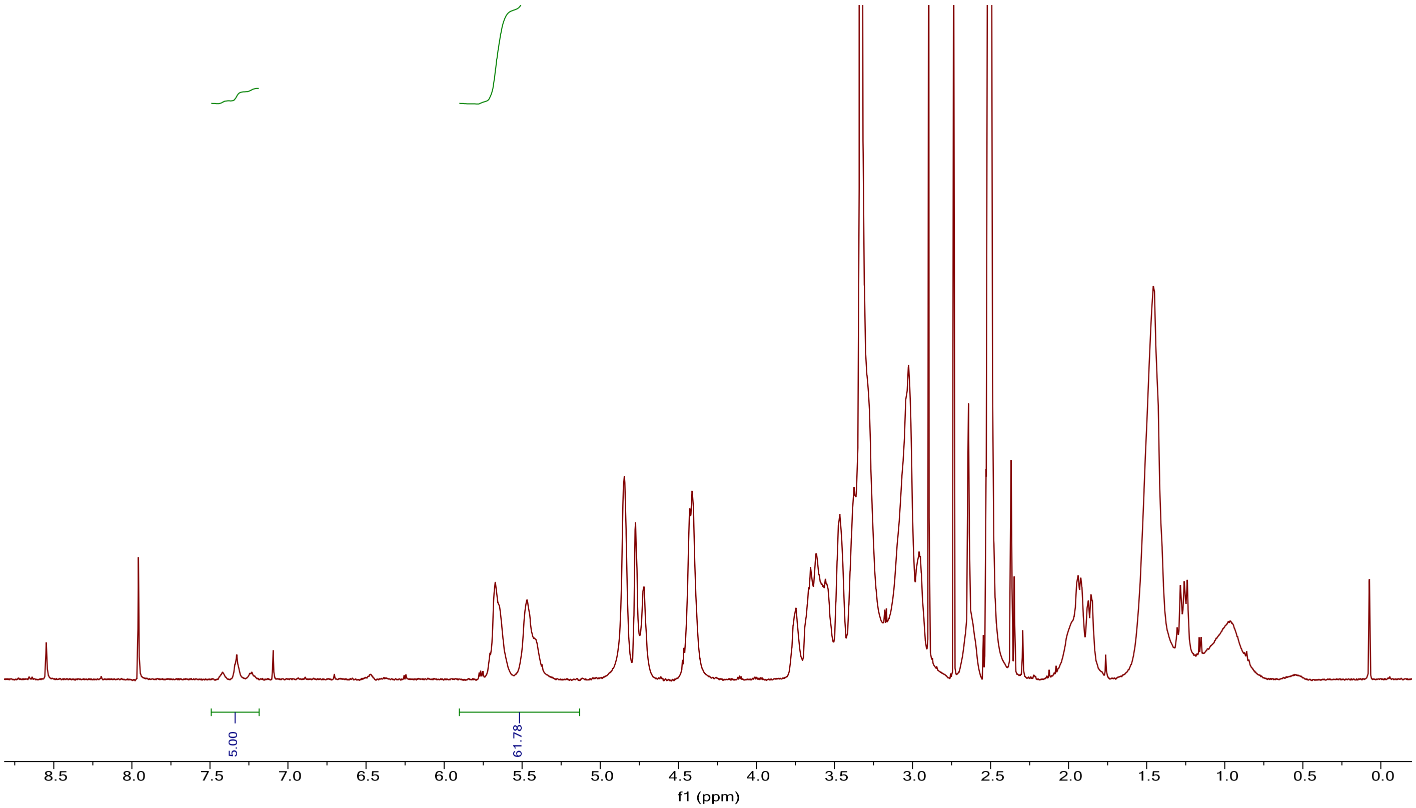

^{1}H-NMR spectra of **polyPEG** in DMF-*d7*:

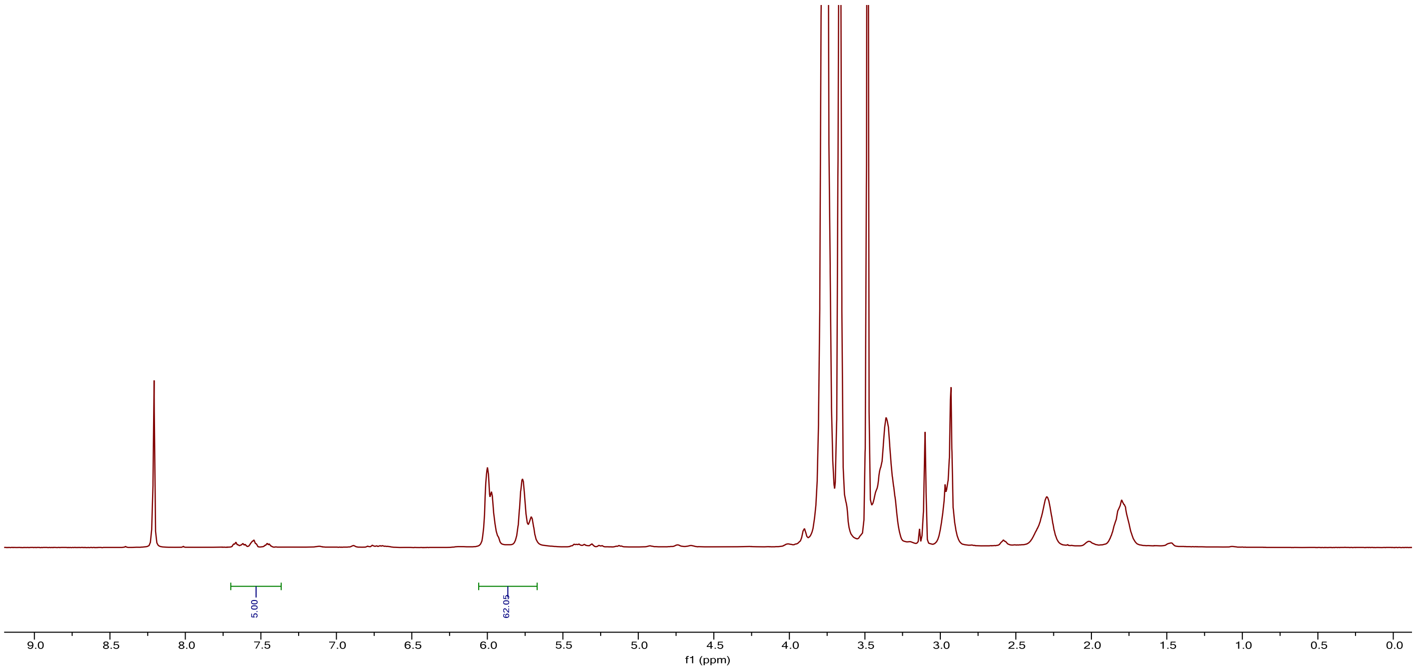

9. Supplemental References

(1) Jeon, S.; Haynie, T.; Chung, S.; Callmann, C. E. Bioinspired, Carbohydrate-Containing Polymers Efficiently and Reversibly Sequester Heavy Metals. *ACS Cent Sci* **2024**, *10* (9), 1782-1788. DOI: 10.1021/acscentsci.4c01010

(2) Jeon, S.; Odom, T. L.; Williams, C. A.; Callmann, C. E. Glycopolymer-Mediated Selective Separation of Middle Rare Earth Elements. *Angew Chem Int Ed Engl* **2025**, *64* (6), e202417505. DOI: 10.1002/anie.202417505
